# Clerodane Diterpenes from the Marine Sponge *Raspailia bouryesnaultae* Collected in South Brazil

**DOI:** 10.3390/md17010057

**Published:** 2019-01-16

**Authors:** Cintia Lhullier, Eliane de Oliveira Tabalipa, Fernanda Nienkötter Sardá, Louis Pergaud Sandjo, Naira Fernanda Zanchett Schneider, João Luis Carraro, Cláudia Maria Oliveira Simões, Eloir Paulo Schenkel

**Affiliations:** 1 Laboratório de Produtos Naturais, Departamento de Ciências Farmacêuticas, Universidade Federal de Santa Catarina, Florianópolis 88040-900, SC, Brazil; elianetabalipa@yahoo.com.br (E.d.O.T.); fernandasarda@gmail.com (F.N.S.); eloirschenkel@gmail.com (E.P.S.); 2 Laboratório de Farmacognosia, Departamento de Ciências Farmacêuticas, Universidade Federal de Santa Catarina, Florianópolis 88040-900, SC, Brazil; p.l.sandjo@ufsc.br; 3 Laboratório de Virologia Aplicada, Departamento de Ciências Farmacêuticas, Universidade Federal de Santa Catarina, Florianópolis 88040-900, SC, Brazil; nairafzs@gmail.com (N.F.Z.S.); claudia.simoes@ufsc.br (C.M.O.S.); 4 Departamento de Invertebrados, Museu Nacional, Universidade Federal do Rio de Janeiro, Rio de Janeiro 20940-040, RJ, Brazil; joao.porifera@gmail.com

**Keywords:** *Raspailia bouryesnaultae*, sponge, clerodane diterpenes, antiproliferative effects, anti-herpes activity

## Abstract

The marine sponge *Raspailia bouryesnaultae*, collected in South Brazil, was selected for detailed investigation considering the results of a screening that pointed to an in vitro antiproliferative effect against non-small cells of human lung cancer (A549) and anti-herpes activity against *Herpes Simplex* *virus* type 1 (KOS and 29R strains) of ethanolic extracts. The fractionation and chemical investigation of the sponge’s hexanic fraction led to the isolation and structural elucidation of six clerodane diterpenes. The main component was identified as the already-reported raspailol (**1**), isolated from a sponge of the same genus collected in New Zealand. The structure of a new diterpene (**2**) with a rearranged skeleton was established by high-resolution mass spectrometry (HRMS) and 1D and 2D Nuclear magnetic resonance spectroscopy (NMR) experiments, and named here as raspadiene. Furthermore, four diterpenes were elucidated as isomers of clerodane diterpenes previously obtained from plants, namely kerlinic acid (**3**), kerlinic acid methyl ester (**4**), annonene (**5**), and 6-hydroxyannonene (**6**). They differ in their stereochemistry, since these diterpenes are characterized by a *trans* ring fusion at the decalin moiety and the relative configuration of the two methyl groups at C-8 and C-9 in a *cis* relationship (type *trans/cis*). The *Raspailia* diterpenes have a *cis* ring fusion at the decalin moiety, and the two methyl groups at C-8 and C-9 are in a *trans* relationship (type *cis*/*trans*). The isolated compounds were evaluated for their potential antiproliferative effects on human cancer cell line A549, and it was observed that the diterpenes bearing a hydroxyl group at C-6 exhibited moderate cytotoxic activity, with 50% inhibitory concentration (IC_50_) values lower than 25 μM. The evaluation of the potential anti-herpes activity against *Herpes Simplex Virus* type 1 (HSV-1, KOS and 29R strains) showed that the more promising results were observed for the new compound **2**, since it inhibited HSV-1 (KOS and 29R strains) replication by 83% and 74%, respectively.

## 1. Introduction

Sponges (phylum Porifera) are primitive filter-feeders, and although about 9000 species have already been described, it is thought that there are more than 20,000 marine sponges. They can produce high levels of cytotoxic compounds, with the majority coming from the secondary metabolism, protecting them against predation, overgrowth by fouling organisms, and/or competition for space [1]. It was proved that marine sponges produce an enormous array of antitumor, antiviral, anti-inflammatory, immunosuppressive, antibiotic, and other bioactive molecules that have potential for therapeutic use. Many bioactive metabolites from sponges proved to be inhibitors of certain enzymes, which often mediate or produce mediators of intracellular or intercellular messengers involved in disease pathogenesis [2,3,4].

The genus *Raspailia* contains more than 100 described species. These species are distributed world-wide, mainly in shallow waters [5]. Nowadays, over forty different secondary metabolites have been reported from *Raspailia* sponges, belonging to five main families of compounds: Raspailynes, raspailols, asmarines, agminosides, and clerodane diterpenes [6]. The seven compounds isolated in this work, from marine sponge *Raspailia bouryesnaultae*, belong to the class diterpenes of clerodane type. 

Clerodane diterpenes are a large group of naturally occurring secondary metabolites found in hundreds of plant species from various families and described in a few marine sources, like sponges belonging to the genus *Agelas* [7], and especially to the genus *Raspailia,* such as the compounds raspailol [8], raspailenones A and B, raspailodanes A–G, and topsentanes A–B described for *Raspailia topsenti* [9]. During the last 25 years, more than 1300 diterpenoids and norditerpenoids with the clerodane carbon skeleton have been isolated, and recently, Li and co-workers proposed a classification comprising seven types of molecules based on this basic skeleton [7].

The Brazilian shallow-water sponge species *Raspailia bouryesnaultae*, class Demospongiae, is a recently re-described species [10]. In the literature, no chemical studies were found for this species. On the other hand, a cytotoxic activity was reported for the organic extract of the sponge *Raspailia elegans* (currently *Raspailia bouryesnaultae*) [11]. Therefore, in this report we describe the isolation and structure elucidation of one new compound (**2**) and five other diterpenes (**1**, **3**–**6**), and the assessment of the in vitro activity of compounds **1** and **2** against Herpes simplex virus type 1, KOS and 29R strains.

## 2. Results and Discussion

### 2.1. Chemistry

Specimens of sponge *Raspailia bouryesnaultae* collected in the Coral and Aranhas Islands were extracted by maceration with ethanol. The extracts were concentrated under reduced pressure, and the resulting dried extract was suspended in distilled water and partitioned three times with n-hexane.

**Figure 1:**
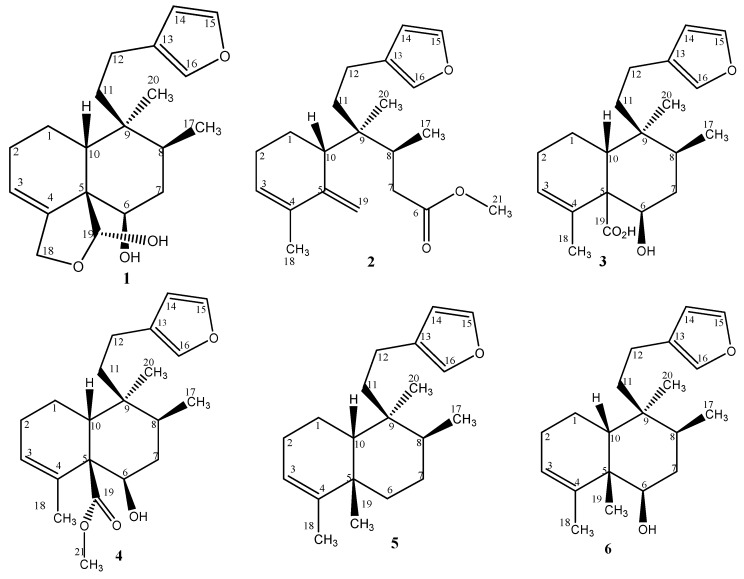
Chemical structures of compounds **1**–**6**.

The hexane fractions were subsequently subjected to a series of chromatographic separations, resulting in the isolation of compounds **1**–**6** (Figure 1). Both collections contained identical compounds, but in different ratios. The major component was **1** in both extracts. All compounds were isolated as colorless oils. 

Compound **1**—The molecular formula was deduced as C_20_H_28_O_4_ by the NMR data and by the high-resolution mass spectrum that showed a pseudo-molecular ion peak [M + Na]^+^ at *m*/*z* = 355.1878 (calculated *m*/*z* 355.1880). Detailed analysis of the ^1^H and ^13^C NMR data, as shown in Table 1; Table 2, revealed the following substructures: A β-monosubstituted furan ring indicated by characteristic ^1^H NMR resonances at δ_H_ 7.33, 7.18, and 6.27 and the ^13^C NMR resonances at δ_C_ 142.6, 138.3, and 111.1; an olefinic methine (δ_H_ 5.76 and δ_C_ 120.7); a carbinol methine (δ_H_ 3.84 and δ_C_ 74.6); an acetal methine resonance at δ_H_ 5.64 and δ_C_ 99.6, and an oxygenated methylene at δ_C_ 68.7 (δ_H_ 4.38 and 4.48, *J* = 11.7 Hz). A methyl singlet could be observed at δ_H_ 0.95 and δ_C_ 25.3, and a methyl doublet at δ_H_ 0.91 (*J* = 6.8 Hz)/δ_C_ 15.7. The presence of only two methyl groups and a further five methylene groups suggested the structure of a diterpene containing a decalin moiety, bounded by two methylene groups to the monosubstituted furan ring, as commonly observed in diterpenes [7]. Considering the seven double-bond equivalents, an additional ring was deduced by the presence of an acetal methine resonance and an oxygenated methylene. These data pointed to the structure of a previously described clerodane diterpene, isolated from a sponge of the same genus (*Raspailia* sp.) collected and studied in New Zealand [8], named raspailol (Figure 1). Careful comparison of the reported spectral data confirmed this structure, which is characterized by the presence of a 5:10 *cis* ring junction at the decalin moiety, and also by the relative stereochemistry at C-8 and C-9, with the two methyl groups in a *trans* relationship, therefore classified as clerodane of the type *cis*/*trans* [7]. 

**Figure 2:**
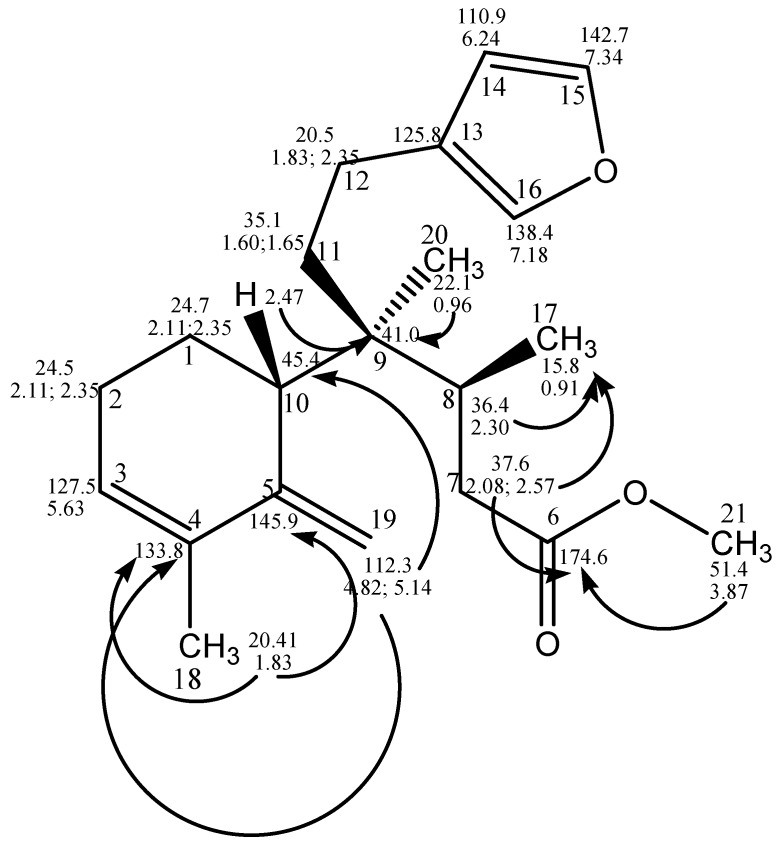
Selected HMBC correlations observed for compound **2**.

Compound **2**—The molecular formula was deduced from the NMR spectral data and the high resolution mass spectrum as C_21_H_30_O_3_ (molecular peak [M + H]^+^ at *m*/*z* 331.2272; calculated 331.2268), pointing to seven degrees of unsaturation. The ^13^C NMR spectrum showed 21 carbon signals, as shown in Table 1 and Figure S3, including five quaternary carbons, six methylene groups, six methine groups, and four methyl groups, which were assigned by a HSQC/DEPT spectrum (Figure S3 in Supplementary Materials). The presence of a β-monosubstituted furan ring was indicated by the characteristic ^1^H NMR resonances at δ_H_ 7.34, 7.18, and 6.27 and the corresponding ^13^C NMR resonances at δ_C_ 142.7 (C-15), 138.4 (C-16) and 110.9 (C-14). HMBC and COSY experiments revealed the attachment of two methylene groups (C-12, δ_C_ 20.5; C-11, δ_C_35.1) to the furan ring at the substituted carbon (C-13, δ_C_ 125.8) [9]. The comparison of the spectroscopic data (^1^H and ^13^C NMR) of **2** with compound **1** (raspailol) pointed to the absences of the acetal methine resonance (at δ_C_ 99.6), the oxygenated methylene (at δ_C_ 68.7), and the carbinol methine resonance at δ_H_ 3.84 (H-6, δ_C_ 74.6). Instead, the spectra clearly showed the presence of an exocyclic methylene, indicated by the resonances in δ_H_ 5.14 and 4.82 with corresponding carbons at δ_C_ 112.3 and δ_C_ 145.9 (quaternary), and also the presence of an oxygenated carbon as an ester group, as indicated by the ^13^C NMR signal at δ_C_ 174.6, and the IR absorption bands 1739 cm^−1^. These data suggested that in the main compound of the extract raspailol, the C-19 hemiacetal ring was opened, forming the exocyclic methylene group bounded to ring A, with bond rupture between C-5 and C-6. In this process, the carbinol subunit of (**1**) was oxidized and esterified, resulting in the carboxymethyl group at position 6. The opening of the hemiacetal ring in the structure of raspailol was already pointed out by the authors that reported its first isolation, where they mentioned the probable reversible ring opening of the hemiacetal ring to an aldehyde [8]. This new rearranged skeleton could be confirmed by the detailed observation of the HMBC correlation corresponding to ring A, between the hydrogens at δ_H_ 5.14 and 4.82 with the quaternary carbons in δ_C_ 133.8 (C-4), and between the methyl group singlet in δ_H_ 1.83 (H–C-18) with the quaternary carbons in δ_C_ 133.8 (C-4) and 154.9 (C-5). The other atoms of the former ring B (C-7, C-8, and C-9), as well the quaternary (C-20) and tertiary (C-17) methyl groups, could be located in this new structure by the HMBC correlations, according to Figure 2. The relative configurations at C-8 and C-9 were assumed to be the same as in raspailol, as this part of the moiety was not affected by the bond rupture between C-5 and C-6. Based on the above-mentioned data, metabolite **2** was identified as a new clerodane derivative, named here as raspadiene, possessing the structure proved to be as shown in Figure 2.

Compound **3**—The high-resolution mass spectrum displayed the pseudo-molecular peak at *m*/*z* = 333.2029 ([M + H]^+^), indicating the molecular formula C_20_H_28_O_4_, which corresponds to seven double-bond equivalents. The NMR spectroscopic features of **3**, given in Table 1 and Table 2, showed close resemblance with those of raspailol (compound **1**), disclosing the presence of a β-monosubstituted furan ring, an olefinic methine identified by NMR resonances δ_H_ 5.88 with corresponding carbon at δ_C_ 129.1 (C-3), and a carbinol signal (^1^H NMR at δ_H_ 3.86 with corresponding carbon at δ_C_ 75.3). As in compound **2**, it was also observed that the acetal methine resonance (at δ_C_ 99.6) and the oxygenated methylene (at δ_C_ 68.7) were absent, and the presence of a carboxylic acid was deduced by ^1^H NMR and ^13^C NMR resonances (δ_H_ 9.51 and δ_C_ 186.8). These resonances were in coherence with IR absorption bands at 1680 and 1180 cm^−1^, and a large absorption band over 3000 cm^−1^, as expected for carboxylic acids. One quaternary methyl group at δ_H_ 0.87/δ_C_ 21.3, one tertiary methyl group at δ_H_ 0.91/δ_C_ 15.6, and another methyl group at δ_H_ 1.88/δ_C_15.6 were located, as in raspailol, at C-9, C-8, and C-4, and corresponded to C-20, C-17, and C-18. Comparison of spectroscopic data of compound **3** with those of previously reported clerodane diterpenes showed similar structural features to kerlinic acid, a substance isolated from the plant species *Salvia keerlii* [12]. Both compounds have the same molecular formula and substituents, but compound **3** is characterized by the *cis* ring fusion at the decalin moiety. Kerlinic acid also differs from compound **3** in the relative stereochemistry at C-8 and C-9, presenting the two methyl groups in a *cis* relationship. The ^13^C NMR chemical shifts have been used to distinguish between the relative configurations of the *trans* or *cis* junctions of the fused rings, and also their relative configurations at C-8 and C-9 [8,9,13]. The detailed comparison of the ^13^C NMR spectral data revealed similarities for the most resonances signals, but significant differences in the values for C-5 (δ_C_ 59.4 in compound **3** and δ_C_ 53.7 in kerlinic acid), C-10 (δ_C_ 43.7 in compound **3** and δ_C_ 48.4 in kerlinic acid), and for C-20 (δ_C_ 21.3 in compound **3** and δ_C_ 15.1 in kerlinic acid). These differences can be explained by the different stereochemistry, for example, the C-20 in kerlinic acid resonances at δ_C_ 15.1, being more protected due to the relative *cis*-*cis* position between the methyl groups at C-8 and C-9. For compound **3,** the expected relative configuration of the methyl groups at C-8 and C-9 is *trans*, as indicated by the spectral data, and also according to the configuration of all other diterpenes clerodanes reported until now for the genus *Raspailia* sp. [8,9].

Compound **4**—The molecular formula C_21_H_30_O_4_ was deduced from the HRMS and NMR data. The high-resolution mass spectrum showed a molecular ion peak [M + Na]^+^ at *m*/*z* 369.2035. The elemental composition accounted for seven double-bond equivalents. The ^1^H and ^13^C NMR data were very similar to that of compound **3**, except for the resonances corresponding to a carboxylic acid group (δ_H_ 9.51 and δ_C_ 186.8) that were not observed. The presence of a methyl ester group was deduced by the resonance at δ_C_ 178.5, with the methoxyl singlet resonating at δ_H_ 3.50 and corresponding carbon at δ_C_ 52.0. The position of the ester group at C-19 was established by correlation in HMBC experiments. Therefore, the structure (Figure 1) was deduced as the methyl ester of compound **3**, and it is not possible to exclude that it is an artefact, as methanol was used in the purification process by column chromatography on Sephadex^R^. 

**Figure 3:**
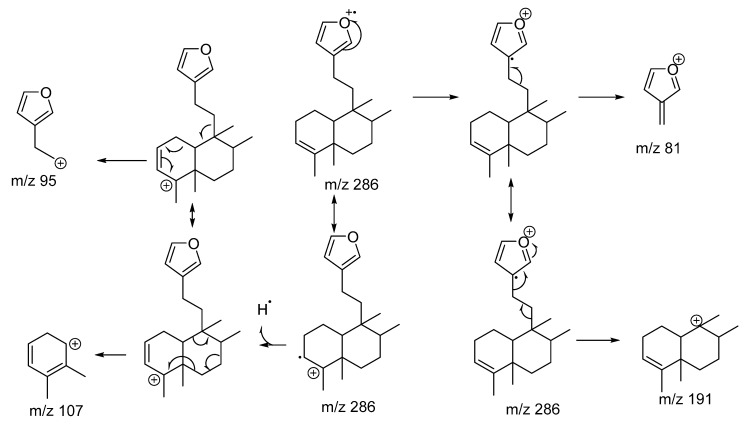
The main fragments observed in the Electron Impact Mass Spectrum of compound **5**.

Compound **5**—The molecular formula C_20_H_30_O was deduced from the Electron Impact Mass Spectrum (EIMS) and NMR data, indicating six degrees of unsaturation. The mass spectrum exhibited a weak radical ion peak [M]^+^ at *m*/*z* 286 (Figure S22). The ^13^C NMR spectrum and the DEPT experiments revealed the presence of 20 carbon atoms, corresponding to four quaternary carbons, six methines, six methylenes, and four methyl groups. The NMR spectroscopy, given in Table 1 and Table 2, showed close resemblance with those of metabolites **3** and **4**, disclosing the presence of the same β-monosubstituted furan ring and an olefinic methine. The ^1^H NMR spectrum further included four methyl groups at δ_H_ 1.98, 1.55, 1.07, and 0.81 and corresponding carbons at δ_C_ 20.1, 37.8, 26.0, and 15.3, which were located at C-18, C-19, C-20, and C-17, respectively. All the positions were confirmed by the 1H-1H-COSY and HMBC experiments (Figures S27 and S28). By consulting the available literature about clerodane diterpenes, it was verified that the ^1^H and ^13^C NMR data of compound **5** were similar to those of annonene, a diterpene previously isolated from *Annona coriacea* [14]. The observed fragmentation in the EIMS, as shown in Figure 3, is in accordance with this deduced structure. However, in annonene, as in the previously discussed kerlinic acid, rings A and B are *trans* fused, and the methyl groups at C-17 and C-20 have a cis relationship. Here in compound **5**, the atoms connected to the C-5 and C-10 carbons, at the junction of the decalinic ring, assume a *cis* position, and the C-17 and C-20 methyl groups are in the *trans* position, as with compounds **1**, **2**, **3**, and **4**, and also according to the stereochemistry reported for the other clerodane diterpenes isolated for the genus *Raspailia* sp. [8,9]. 

Compound **6**—The high-resolution mass spectrum allowed us to establish the molecular formula C_20_H_30_O_2_. The mass spectrum exhibited molecular ion peaks [M + H]^+^ at *m*/*z* 303.2317 (calculated 303.2319). The molecular formula indicates six degrees of unsaturation. The NMR spectroscopic data of **6**, given in Table 1 and Table 2, showed close resemblance with those of compound **5**. The main difference was an additional signal corresponding to a carbinol (δ_H_ 3.98, with corresponding carbon at δ_C_ 75.7). The location of the additional hydroxyl group was established considering the correlations of H–C-6 in the HMBC experiments with the quaternary carbon at δ_C_ 47.1 (C-5). By comparing the data obtained with the literature, it was verified that compound **6** has the same molecular formula and substituents as 6-hydroxyannonene, a clerodane diterpene first isolated from *Croton sonderianus* [15], which is characterized by the *trans* ring fusion at the decalin moiety, and presenting the two methyl groups at C-8 and C-9 in a *cis* relationship. As in compounds **1**, **2**, **3**, and **4**, the observed NMR data for compound **6** pointed to the *cis* ring fusion at the decalin moiety and the *trans* position of the methyl groups at C-17 and C-20, which is also in accordance with the stereochemistry reported for the other clerodane diterpenes isolated for the genus *Raspailia* sp. [8,9].

### 2.2. Antiproliferative Effects

A preliminary screening of the potential antiproliferative effects on human lung cancer A549 cells performed with the ethanolic extracts and hexane fractions of *Raspailia bouryesnaultae*, collected in the Coral and Aranhas Islands, showed that the hexane fraction from the Coral Island exhibited weak activity, with a 50% inhibitory concentration (IC_50_) value of 219.2 μg/mL, and the hexane fraction from the Aranhas Island presented medium activity, with an IC_50_ value of 89.3 μg/mL. 

Compounds **1**–**6** were screened for their potential antiproliferative effects on human lung cancer cell line A549 and the non-tumor Vero cell line to estimate their selectivity. The results of the cytotoxicity assessment of the tested compounds after 48 h of incubation are given in Table 3. The lowest activity against A549 cells was observed for compound **5**, which consists of the clerodane diterpene skeleton without any functional groups. The activity increased when functional groups were introduced to this basic skeleton, and compounds **1**, **4**, and **6**, bearing a hydroxyl group at C-6, exhibited moderate cytotoxic activity, with IC_50_ values lower than 25 μM. Compounds **1** and **4** were the most selective for the cancer cell line over the non-tumor cell line.

Clerodane diterpenes have been largely investigated concerning their cytotoxic activity. The extensive review of Li and collaborators [7] described a variety of clerodane derivatives with cytotoxic activity against many cancer cell lines. Other clerodanes with similar stereochemistry (considering the 5:10 *cis* junction of the fused rings of the decalin moiety and the relative stereochemistry at C-8 and C-9) were reported to be active in the 1–10 µM range. However, these more active clerodanes presented an unsaturated open side chain at C-9, instead of the furanyl ethyl side chain as in the compound reported here [16,17].

### 2.3. Anti-Herpes Activity

Compounds **1**–**6** were also screened for their potential anti-herpes activity against *Herpes Simplex Virus* type 1 (HSV-1, KOS and 29R strains, sensitive and resistant to acyclovir, respectively). The results indicated that compounds **2** and **4** inhibited viral HSV-1 (KOS strain) replication by 83% and 50%, respectively, and compound **2** also inhibited HSV-1 (29R strain) replication by more than 70%. The other compounds did not present significant inhibition of virus replication (<50%). The results are show in Table 4.

In view of these results, compounds **2** and **4** were further assayed to determine their IC_50_ and selective index (SI) values. The results showed that compound **2** demonstrated the best activity, with SI values >3.0 for both tested strains. These results are given in Table 5.

Both active compounds (**2** and **4**) possessed a carboxymethyl ester group, whereas the other ones (**1** and **3**) without this specific group displayed weak activity or were not active. The possible structure–activity relationships remain to be investigated based on a higher extensive panel of compounds.

## 3. Materials and Methods

### 3.1. General

Infrared spectra (IR) were obtained in an IR Prestige-21 FTIR-8400 S (Shimadzu, Tokyo, Japan) using KBr. UV spectra were obtained on a UV/Vis lambda 15 spectrophotometer (Perkin-Elmer, Waltham, MA, USA)) using ethanol. NMR spectra were obtained on a Bruker Fourier 300 (^1^H: 300 MHz, ^13^C: 75 MHz) and DRX-400 (^1^H: 400 MHz, ^13^C: 100 MHz) in CDCl_3_. Chemical shifts were given in δ (ppm), using tetramethylsilane (TMS) as an internal standard. The 2D experiments (HSQC, HMBC, 1H-1H COSY, NOESY) were performed using standard Bruker pulse sequences. Low-resolution Electron Impact mass spectra were recorded on a Gas chromatography–mass spectrometry system, consisting of a Perkin-Elmer GC Clarus 680^®^ coupled to a Clarus SQ8 mass spectrometer with a quadrupole detector (scan range 55–550 Da), using an Elite-5mS non-polar silica capillary column (30 m, 0.25 mm i.d., 0.25 μm film thickness). The samples (1 µL) were introduced via an all-glass injector working in the split mode (5.0 mL min^−1^), with helium (99.999% purity) as a carrier gas at a flow rate of 1 mL min^−1^. The ion source temperature was 180 °C, and the GC inlet and transfer line to the ion source were both held at 250 °C.

High-resolution mass spectra were recorded by direct sample injection on a mass spectrometer Waters^®^ Xevo G2-S QTof (Waters, Milford, MA, USA) equipped with an electrospray (ESI) probe which can operate in positive and negative mode ionization. ESI positive mode ionization was used with capillary voltage of 2 kV. The temperatures of the cone and desolvation were set at 80 and 120 °C, respectively. N2 flows to control the temperatures of the cone and the desolvation were 100 and 400 L/h, respectively. Leucine encephalin was the lockspray reference sample, with reference mass value at *m*/*z* 556.2771.

During the isolation procedures, chromatographic separations were performed using a medium-pressure liquid chromatography system (Syncore^®^/Büchi, Buchi, New Castle, DE, USA)) and gravity column chromatography on silica gel 60 (0.040–0.063 mm, Vetec^®^, St. Louis, MO, USA)). Thin Layer Chromatography analysis was performed on silica gel F254 plates (Macherey-Nagel^®^, Düren, Germany)), and spots were detected after spraying with anisaldehyde sulfuric reagent and heating at 100 °C for 1 min. HPLC separations were conducted using a Shimadzu HPLC System with LC-10AD pump and SPD-10A UV/vis, using a Luna C_18_ (Phenomenex^®^, Torrance, CA, USA, 250 cm × 10 mm, 10 µm) column.

### 3.2. Biological Material Collection

Specimens of *Raspailia bouryesnaultae* were collected by SCUBA at a depth of 10–12 m off the coast of Coral Island (27°54′28″S; 48°31′12″W) in May 2014, Garopaba, and off the coast of Aranhas Island (27°29′12″S; 48°21′37″W) in May 2016, Florianópolis, Santa Catarina State, South Brazil. The biological materials were kept frozen at −20 °C. Voucher specimens were identified by João Luis Carraro, and were deposited at the Sponge Collection of the Departamento de Invertebradostes (Universidade Federal de Rio de Janeiro, Rio de Janeiro, RJ, Brazil).

### 3.3. Extraction and Isolation

The frozen specimens of sponge from Coral Island (2.0 kg) were extracted three times with ethanol, for seven days each time. The joined extracts were concentrated under reduced pressure. The resulting dried extract (6.5 g) was suspended in distilled water and partitioned three times with n-hexane, resulting in 3.3 g of n-hexane fraction (HF fraction). The HF fraction was subjected to a medium-pressure liquid chromatographic column on silica gel (hexane-ethyl acetate-methanol gradient) to afford 20 fractions (A–T). Fractions FC, FH, and FP yielded compound **2** (3.5 mg), compound **4** (7.3 mg), and compound **1** (32.7 mg), respectively. Fractions FF, FG, and FJ (128 mg) were purified by reverse-phase HPLC, using MeOH as an eluent, to afford compounds **4** (22 mg) and **6** (6.4 mg). Fractions FB and FE (484 mg) were combined and further fractionated by gravity column chromatography on silica gel, using cyclohexane with increasing amounts of EtOAc as the mobile phase, to yield 12 fractions (E1–E12). Fractions E3 and E5 (35 mg) were purified by reverse-phase HPLC, using MeOH:H_2_O (9:1) as an eluent, to afford compound **2** (6.6 mg). Fraction FI (7.9 mg) was purified by reverse-phase HPLC, using MeOH as an eluent, to afford compound **3** (3.2 mg).

The frozen specimens of sponge from Aranhas Island (2.0 kg) were extracted three times with ethanol, for seven days each time. The joined extracts were concentrated under reduced pressure. The resulting dried extract (6.5 g) was suspended in distilled water and partitioned three times with n-hexane, resulting in 3.3 g (HF fraction). The HF fraction was subjected to a medium-pressure liquid chromatographic column on silica gel (hexane-ethyl acetate-methanol gradient) to afford 17 fractions (A–Q). Fraction FB yielded compound **5** (5.7 mg). Fraction FM yielded compound **1** (17.6 mg). Fractions FH, FJ, and FL (69.3 mg) were purified by reverse-phase HPLC, using MeOH as an eluent, to afford compounds **4** (6.3 mg) and **3** (2.9 mg). Fractions FC, FE, and FF (194.5 mg) were combined and further fractionated by gravity column chromatography on silica gel, using cyclohexane with increasing amounts of EtOAc as the mobile phase, to yield 10 fractions (E1–E10). Fractions E3, E4, and E5 (15 mg) were purified by reverse-phase HPLC, using MeOH:H_2_O (9:1) as an eluent, to afford compound **2** (2.5 mg).

**Compound 1 (raspailol):** Colorless oil, HRMS [M + Na]^+^ *m*/*z* = 355.1878 (calculated for C_20_H_28_O_4_ = 355.1880). NMR: Table 1 and Table 2 and Supplementary Information.

**Compound 2 (raspadiene):** Colorless oil, IR (KBr) υ cm^−1^: 2910, 1735, 1620, 1440, 1285, 1140, 1015. HRMS *m*/*z*: 331.2272 [M + H]^+^ (calculated for C_21_H_31_O_3_ = 331.2268). NMR: Table 1 and Table 2 and Supplementary Information. 

**Compound 3:** Colorless oil, IR (KBr) υ cm^−1^: 3520, 2910, 2380, 1680, 1540, 1510, 1180, 880, 620. HRMS *m*/*z*: 333.2029 [M + H]^+^ (calculated for C_20_H_29_O_4_: *m*/*z* = 333.2060). NMR: Table 1 and Table 2 and Supplementary Information.

**Compound 4:** Colorless oil, IR (KBr) υ cm^−1^: 3510, 2920, 1740, 1680, 1560, 1240, 1180, 1080, 1060, 870. HRMS *m*/*z*: 369.2035 [M + Na]^+^ (calculated for C_21_H_30_O_4_Na = 369.2036). NMR: Table 1 and Table 2 and Supplementary Information.

**Compound 5:** Colorless oil, IR (KBr) υ cm^−1^: 3420, 1640, 1580, 1540. EIMS (70 eV): 286 (M, 5%), 207 (100%), 191 (20%), 107 (12%), 95 (31%), 81 (35%) NMR: Table 1 and Table 2 and Supplementary Information.

**Compound 6:** Colorless oil, IR (KBr) υ cm^−1^: 3510, 1620, 1440, 1380, 620. HRMS *m*/*z*: 303.2317 [M + H]^+^ (calculated for C_20_H_31_O_2_ = 303.2319). NMR: Table 1 and Table 2 and Supplementary Information.

### 3.4. Antiproliferative Assays

Non-small cells of human lung cancer cells (A549; ATCC: CCL 195) and African Green monkey kidney fibroblasts (Vero; ATCC: CCL81) were grown in Eagle’s minimum essential medium (MEM; Cultilab, Campinas, Brazil) supplemented with 1% glutamine (Gibco, Carlsbad, CA, USA) and 10% fetal bovine serum (FBS; Gibco, Carlsbad, CA, USA), and maintained at 37 °C and 5% CO_2_ in a humidified atmosphere. The antiproliferative screening was performed by sulforhodamine B assay (SRB) [18]. In summary, A549 cells were seeded into 96-well plates (2.5 × 10^4^ cells per well) and exposed to different concentrations of compounds **1**–**6** for 48 h. Paclitaxel (PAC, Sigma-Aldrich, St. Louis, MO, USA) was used as a positive control. After incubation, 10% trichloroacetic acid (TCA) was added to each well to fix the cells, and then the cells were washed and stained with SRB. The protein-bound dye was dissolved in 10 mM Tris-Base [(tris(hydroxymethyl) aminomethane] solution, and the optical densities (OD) were read at 510 nm on a Spectra Max M2 spectrophotometer (Molecular Devices, Molecular Devices, San Jose, CA, USA). The 50% inhibitory concentration (IC_50_) of each compound was defined as the concentration that inhibited cell viability by 50% when compared to untreated controls. Vero cells were seeded into 96-well plates (2.5 × 10^4^ cells per well) to determine, also by SRB assay, the CC_50_ of each compound, which is defined as the concentration that reduced non-tumor cell viability by 50% when compared to untreated controls. The ratio between CC_50_ and IC_50_ values was calculated to obtain the selectivity index (SI) of each sample.

### 3.5. Anti-Herpes Assays

Firstly, the cytotoxicity of compounds **1**–**6** was determined by SRB assay as described above. HSV-1 (KOS and 29R strains, which are sensitive and resistant to acyclovir, respectively, Faculty of Pharmacy, University of Rennes I, Rennes, France) were propagated in the permissive Vero cells. Viral stocks were titrated as based on plaque-forming units (PFU), counted by plaque assay as previously described [19], and stored at −80 °C until used. To perform the anti-HSV-1 screening, the viral plaque number reduction assay was used as described previously by Boff et al. [20]. Briefly, confluent Vero cell monolayers (2.5 × 10^5^ cells per well) were infected with approximately 100 PFU of each virus strain for 1 h at 37 °C. Treatments were performed by adding non-cytotoxic concentrations of the compounds after viral infection (post-infection treatment). Cells were then washed with phosphate-buffered saline (PBS) and overlaid with MEM containing 1.5% carboxymethylcellulose (CMC; Sigma-Aldrich) in the presence or absence of different concentrations of the compounds and incubated for 48 h. Cells were fixed and stained with naphthol blue-black (Sigma-Aldrich), and viral plaques were counted by using a stereomicroscope. The results were presented as percentages of viral inhibition or by IC_50_ values, which are defined as the concentration of each sample that inhibited viral plaque formation by 50% (IC_50_) when compared to untreated controls. The ratio between CC_50_ and IC_50_ values was calculated to obtain the selectivity index (SI) of each sample. Acyclovir (ACV, Sigma-Aldrich) was used as a positive control for HSV-1 (KOS strain) and as a negative control for HSV-1 (29-R strain) replication.

## 4. Conclusions

Chemical investigation of the sponge *Raspailia bouryesnaultae* collected from South Brazil led to the isolation and structural elucidation of raspailol as the main component, previously reported from a sponge of the same genus collected in New Zealand, and a new diterpene with a rearranged skeleton. 

Furthermore, four diterpenes were elucidated as isomers of clerodane diterpenes previously isolated from plants, namely kerlinic acid, kerlinic acid methylester, annonene, and 6-hydroxyannonene. They differ in their stereochemistry, since these diterpenes are characterized by a *trans* ring fusion at the decalin moiety, and the relative configuration of the two methyl groups at C-8 and C-9 in a *cis* relationship type (*trans*/*cis*), whereas the *Raspailia* diterpenes have a *cis* ring fusion at the decalin moiety, and the two methyl groups at C-8 and C-9 are in a *trans* relationship (type *cis*/*trans*).

The isolated compounds displayed antiproliferative effects on human cancer cell line A549, exhibiting moderate cytotoxic activity, with IC_50_ values lower than 25 μM for the diterpenes bearing a hydroxyl group at C-6. 

The evaluation of the potential anti-herpes activity (HSV-1, KOS and 29R strains, sensitive and resistant to acyclovir, respectively) showed that the more promising results were observed for the new compound **2**, since it inhibited HSV-1 (KOS and 29R strains) replication by 83% and 74%, respectively.

## Figures and Tables

**Table 1:** ^13^C NMR data of compounds **1**–**6** in CDCl_3_ (100 MHz; δ in ppm).

Position	1	2	3	4	5	6
1	19.7	24.7	38.2	19.1	19.6	21.6
2	25.8	24.5	18.7	26.5	25.8	27.1
3	120.7	127.5	129.1	124.8	122.4	123.9
4	139.1	133.8	136.2	135.5	148.0	142.1
5	53.4	145.9	59.4	54.6	38.2	47.1
6	74.6	174.6	75.3	75.5	33.0	75.7
7	38.3	37.6	38.5	38.8	25.8	38.6
8	36.8	36.4	36.1	36.8	37.4	37.0
9	38.0	41.0	38.9	38.7	38.4	39.1
10	40.4	45.4	43.7	44.4	44.6	43.5
11	32.8	35.1	26.1	32.1	32.3	46.0
12	18.9	20.5	18.7	18.9	18.9	19.4
13	130.0	125.8	124.9	125.7	123.2	124.4
14	111.1	110.9	110.6	110.9	111.0	111.0
15	142.6	142.7	142.8	142.7	142.6	142.7
16	138.3	138.4	138.8	138.3	138.5	138.5
17	15.7	15.8	15.6	15.7	15.3	15.6
18	68.7	20.4	23.4	22.8	20.1	22.7
19	99.6	112.3	186.8	178.5	37.8	18.1
20	25.3	22.1	21.3	26.4	26.0	28.6
21	-	51.4	-	52.0	-	-

**Table 2:** ^1^H NMR data of compounds **1–6** in CDCl_3_ (400 MHz; δ in ppm; *J* in Hz).

Position	1	2	3	4	5	6
1β	1.73 (m)	2.35 (d, 1.83)	1.76 (m)	1.73 (m)	1.70 (m)	1.71 (m)
2α	2.20 (m)	2.11 (d, 1.83)	1.48 (m)	2.16 (m)	1.95 (m)	2.04 (m)
2β	2.20 (m)	2.35 (d, 1.83)	1.75 (m)	2.16 (m)	2.00 (m)	2.04 (m)
3	5.76 (m)	5.63 (d, 2.8)	5.88 (s)	5.64 (s)	5.35 (s)	5.50 (s)
6α	3.84 (m)	-	3.86 (m)	3.87 (td, 11.8, 4.7)	1.21 (m)	3.98 (m)
6β	-	-	-	-	1.24 (s)	-
7α	1.48 (m)	2.08 (m)	1.62 (m)	1.62 (m)	1.95 (m)	1.56 (m)
7β	1.65 (m)	2.57 (m)	1.76 (m)	1.73 (m)	2.00 (m)	1.75 (m)
8	1.81 (m)	2.30 (m)	1.76 (m)	1.83 (m)	1.51 (m)	2.16 (m)
10	2.00 (dd, 13.2, 3.1)	2.47 (m)	2.16 (m)	2.36 (m)	1.43 (m)	2.20 (d, 7.8)
11α	1.40 (m)	1.60 (m)	2.07 (m)	1.26 (m)	1.49 (m)	1.83 (m)
11β	1.56 (m)	1.65 (m)	2.17 (m)	1.52 (m)	1.61 (m)	2.09 (m)
12α	2.18 (m)	1.83 (d, 1.51)	2.19 (m)	2.36 (m)	2.35 (m)	2.52 (m)
12β	2.61 (td, 12.9, 3.3)	2.35 (d, 1.51)	2.39 (m)	2.45 (m)	2.45 (m)	2.52 (m)
14	6.27 (s)	6.24 (s)	6.25 (s)	6.27 (s)	6.28 (s)	6.27 (s)
15	7.33 (t, 1.6)	7.34 (t, 1.6)	7.36 (t, 1.5)	7.35 (t, 1.6)	7.35 (s)	7.35 (t, 1.6)
16	7.18 (s)	7.18 (s)	7.22 (s)	7.19 (s)	7.21 (s)	7.21 (s)
17	0.91 (d, 6.8)	0.91 (s)	0.91 (d, 6.8)	0.88 (d, 6.5)	0.81 (d, 6.8)	0.85 (d, 6.9)
18α	4.34 (dq, 11.7, 1.9)	1.83 (s)	1.80 (s)	1.76 (s)	1.98 (s)	1.88 (m)
18β	4.48 (m)	-	-	-	-	-
19α	5.64 (d, 2.6)	4.82 (s)	9.5 (d, 2.9)	-	1.55 (s)	1.28 (s)
19β	-	5.14 (s)	-	-	-	-
20	0.95 (s)	0.96 (s)	0.87 (s)	0.97 (s)	1.07 (s)	1.03 (s)
21	-	3.87 (s)	-	3.50 (s)	-	-
6OH	2.92 (ls)	-	-	2.83 (ls)	-	-

**Table 3:** Antiproliferative effects of compounds **1**–**6** on non-small cells of human lung cancer (A549) and Vero (healthy fibroblasts from kidneys of African Green monkeys) cell lines, after 48 h of treatment by sulforhodamine B assay.

Compounds	IC_50_ µM	CC_50_ µM	SI
	A549 Cell Line (Confidence Interval 95%)	VERO Cell Line (Confidence Interval 95%)	
**1**	24.12 (15.68 a 37.09)	148.3 (93.30 a 235.7)	6.14
**2**	100.3 (43.54 a 231.2)	>250	2.49
**3**	66.22 (49.16 a 89.21)	181.5 (140.7 a 234.2)	2.74
**4**	20.63 (11.64 a 36.55)	98.41 (74.08 a 130.7)	4.77
**5**	143.7 (107.7 a 191.9)	>250	>1.74
**6**	24.51 (17.72 a 33.89)	40.77 (27.74 a 59.91)	1.66

Data are presented as IC_50_ values (µM) and 95% confidence intervals obtained by non-linear regression. Data represent the mean of three independent experiments; CC_50_: cytotoxic concentration for 50% of cells; IC_50_: concentration that inhibited 50% of cell viability; SI: selective index = CC_50_/IC_50._

**Table 4:** Percentages of HSV-1 (KOS strain and 29R strain) inhibition after 48 h of treatment with compounds **1**–**6** by viral plaque number reduction assay.

Compounds	Tested Concentration (µg/mL)	% HSV-1 Inhibition ± SD	
		KOS Strain	29R Strain
**1**	100	34.56 ± 7.36	0
**2**	100	83.41 ± 16.12	74.21 ± 6.14
**3**	100	24.42 ± 4.04	32.03 ± 6.12
**4**	50	50.23 ± 8.24	14.45 ± 5.46
**5**	100	15.20 ± 4.65	30.85 ± 9.07
**6**	25	0	0

Data represent the mean of three independent experiments ± SD.

**Table 5:** Anti-herpes activity [anti-HSV-1 (KOS and 29R strains)] after 48 h of treatment with compounds **2** and **4** by viral plaque number reduction assay.

Compounds	CC_50_	IC_50_		SI	
	Vero Cells	(KOS Strain)	(29R Strain)	(KOS Strain)	(29R Strain)
**2**	>250	81.39 ± 9.82	74.93 ± 7.30	>3.07	>3.33
**4**	98.41 (74.08–130.7)	52.38 ± 4.78	ND	1.87	-

HSV-1 = *Herpes Simplex Virus* type 1; Data represent the mean of three independent experiments; CC_50_: cytotoxic concentration for 50% of cells (95% Confidence Interval); IC_50_: concentration ± SD that inhibited 50% of viral replication; SI: selective index = CC_50_/IC_50_; ND: not determined.

## Supplementary Materials

The following are available online at http://www.mdpi.com/1660-3397/17/1/57/s1, Figure S1: High-resolution mass spectrum of compound **1**, Figure S2: ^1^H NMR spectrum of compound **1** at 400 MHz in CDCl_3_, Figure S3: ^13^C NMR spectrum of compound **1** at 100 MHz in CDCl_3_, Figure S4: High-resolution mass spectrum of compound **2**, Figure S5: ^1^H NMR spectrum of compound **2** at 400 MHz in CDCl_3_, Figure S6: ^13^C NMR spectrum of compound **2** at 100 MHz in CDCl_3_, Figure S7: DEPT 135 NMR spectrum of compound **2** at 100 MHz in CDCl_3_, Figure S8: HSQC NMR spectrum of compound **2** at 300 MHz in CDCl_3_, Figure S9: 1H-1H COSY NMR spectrum of compound **2** at 300 MHz in CDCl_3_, Figure S10: HMBC NMR spectrum of compound **2** at 300 MHz in CDCl_3_, Figure S11: High-resolution mass spectrum of compound **3**, Figure S12: ^1^H NMR spectrum of compound **3** at 400 MHz in CDCl_3_, Figure S13: ^13^C NMR spectrum of compound **3** at 75 MHz in CDCl_3_, Figure S14: HSQC NMR spectrum of compound **3** at 300 MHz in CDCl_3_, Figure S15: High-resolution mass spectrum of compound **3**, Figure S16: ^1^H NMR spectrum of compound **4** at 400 MHz in CDCl_3_, Figure S17: ^13^C NMR spectrum of compound **4** at 100 MHz in CDCl_3_, Figure S18: DEPT 135 NMR spectrum of compound **4** at 100 MHz in CDCl_3_, Figure S19: HSQC NMR spectrum of compound **4** at 300 MHz in CDCl_3_, Figure S20: 1H-1H COSY NMR spectrum of compound **4** at 300 MHz in CDCl_3_, Figure S21: HMBC NMR spectrum of compound **4** at 300 MHz in CDCl_3_, Figure S22: High-resolution mass spectrum of compound **5**, Figure S23: ^1^H NMR spectrum of compound **5** at 400 MHz in CDCl_3_, Figure S24: ^13^C NMR spectrum of compound **5** at 100 MHz in CDCl_3_, Figure S25: DEPT 135 NMR spectrum of compound **5** at 100 MHz in CDCl_3_, Figure S26: HSQC NMR spectrum of compound **5** at 300 MHz in CDCl_3_, Figure S27: 1H-1H COSY NMR spectrum of compound **5** at 300 MHz in CDCl_3_, Figure S28: HMBC NMR spectrum of compound **5** at 300 MHz in CDCl_3_, Figure S29: High-resolution mass spectrum of compound **6**, Figure S30: ^1^H NMR spectrum of compound **6** at 400 MHz in CDCl_3_, Figure S31: ^13^C NMR spectrum of compound **6** at 100MHz in CDCl_3_, Figure S32: HSQC NMR spectrum of compound **6** at 300 MHz in CDCl_3_, Figure S33: HMBC NMR spectrum of compound **6** at 300 MHz in CDCl_3_.

## References

[1] Perdicaris S., Vlachogianni T., Valavanidis A. (2013). Bioactive natural substances from marine sponges: New developments and prospects for future pharmaceuticals. Nat. Prod. Chem. Res..

[2] Sagar S., Kaur M., Minnerman K.P. (2010). Antiviral lead compounds from marine sponges. Mar. Drugs.

[3] Vila F.A., Gerwick L. (2010). Marine natural product drug discovery: Leads for treatment of inflammation.cancer.infections and neurological disorders. Immunopharmacol. Immunotoxicol..

[4] Frota M.J., Silva R.B., Mothes B., Henriques A.T., Moreira J.C. (2012). Current status on natural products with antitumor activity from Brazilian marine sponges. Curr. Pharm. Biotechnol..

[5] Hooper J.N.A., Van Soest R.W.M. (2002). SystemaPorifera: A Guide to the Classification of Sponges.

[6] Northover B.J. (2012). Natural Products Studies of Marine Organisms of the South Pacific. Master’s Thesis.

[7] Li R., Morris-Natschke S.L., Kuo-Hsiung L. (2016). Clerodanediterpenes: Sources, structures, and biological activities. Nat. Prod. Rep..

[8] West L.M., Northcote P.T., Battershill C.N. (1998). Two New ClerodaneDiterpenes from the New Zealand Marine Sponge *Raspailia* sp.. Aust. J. Chem..

[9] Ryan. J.M. (2007). Novel Secondary Metabolites from New Zealand Marine Sponges. Ph.D. Thesis.

[10] Lerner C., Carraro J.L., Soest R.V. (2006). Raspailia (Raspaxilla) bouryesnaultae, a new name for Brazilian *Raspaxillaelegans* Boury-Esnault, 1973 (Demospongiae, Poecilosclerida, Raspaileedae) with a redescription and a new record. Zootaxa.

[11] Rangel M., Sanctis B., Freitas J.C., Polatto J.M., Granato A.C., Berlinck R.G.S., Hajdu E. (2001). Cytotocic and neurotoxic activities in extracts of marine sponges (Porifera) from southeastern Brazilian coast. J. Exp. Mar. Bio. Ecol..

[12] Rodríguez-Hahn L., García A., Esquivel B., Cárdenas J. (1987). Structure of kerlinic acid from *Salvia keerlii*. Chemical correlation with melisodoric acid. Can. J. Chem..

[13] Zdero C., Bohlmann F., King R.M. (1991). Diterpenes and Norditerpenes from the Aristeguetia Group. Phytochemistry.

[14] Ferrari M., Pelizzoni F. (1971). New diterpenoids with clerodane skeleton. Pergamon.

[15] Silveira E.R., Mcchesney J.D. (1994). 6,7-Oxygenated neo-clerodane furan diterpenes from *Croton sonderianus*. Phytochemistry.

[16] Itokawa H., Totsuka N., Morita H., Takeya K., Iitaka Y., Schenkel E.P., Motidome M. (1990). New Antitumor Principles, Casearins A-F, for Casearia sylvestris Sw. (Flacourtiaceae). Chem. Pharm. Bull..

[17] Morita H., Nakayama M., Kojima H., Takeya K., Itokawa H., Schenkel E.P., Motidome M. (1991). Structures and Cytotoxic Activity Relationship of Casearins, New Clerodane Diterpenes from Casearia sylvestris SW. Chem. Pharm. Bull..

[18] Vichai V., Kirtikara K. (2006). Sulforhodamine B colorimetric assay for cytotoxicity screening. Nat. Protoc..

[19] Burleson F.G., Chamberts T.M., Wiedbrauk D.L. (1992). Virology: A Laboratory Manual.

[20] Boff L., Silva I.T., Argenta D.F., Farias L.M., Alvarenga L.F., Padua R.M., Braga F.C., Leite J.P., Kratz J.M., Simões C.M.O. (2016). *Strychnos pseudoquina* A. St. Hil.: A Brazilian medicinal plant with promising in vitro anti-herpes activity. J. Appl. Microbiol..

