# Supplementary material for: Clerodane Diterpenes from the Marine Sponge Raspailia bouryesnaultae Collected in South Brazil
Source: Mar Drugs. 2019 Jan 16;17(1):57. doi: 10.3390/md17010057 (PMC6356680; doi:10.3390/md17010057)
Supplement: Supplementary file 1 [file marinedrugs-17-00057-s001.pdf]

Supplementary Information for

Clerodane Diterpenes from the Marine Sponge *Raspailia bouryesnaultae* Collected in South Brazil

Cintia Lhullier[1*+], Eliane de Oliveira Tabalipa[1+], Fernanda Nienkö~~n~~tter Sardá[1], Louis Pergaud Sandjo[2], Naira Fernanda Zanchett Schneider[3], João Luis Carraro[4], Cláudia Maria Oliveira Simões[3] and Eloir Paulo Schenkel[1]

[1] Laboratório de Produtos Naturais, Departamento de Ciências Farmacêuticas, Universidade Federal de Santa Catarina, CEP 88.040-900, Florianópolis, SC, Brazil; E-Mails:lhulliercintia@gmail.com (C.L.); elianetabalipa@yahoo.com.br (E.O.T.); fernandasarda@gmail.com (F.N.S.); eloirschenkel@gmail.com (E.P.S.);

[2]Laboratório de Farmacognosia, Departamento de Ciências Farmacêuticas, Universidade Federal de Santa Catarina, CEP 88.040-900, Florianópolis, SC, Brazil; p.l.sandjo@ufsc.br (LPS)

[3]Laboratório de Virologia Aplicada, Departamento de Ciências Farmacêuticas, Universidade Federal de Santa Catarina, CEP 88.040-900, Florianópolis, SC, Brazil; E-Mails: nairafzs@gmail.com (N.F.Z.S); claudia.simoes@ufsc.br (C.M.O.S.)

[4]Departamento de Invertebrados, Museu Nacional, Universidade Federal do Rio de Janeiro, CEP 20940-040, Rio de Janeiro, RJ, Brazil; E-Mail: joao.porifera@gmail.com (J.L.C)

a) Compound 1 - $C_{20}H_{28}O_4$ (Raspailol)

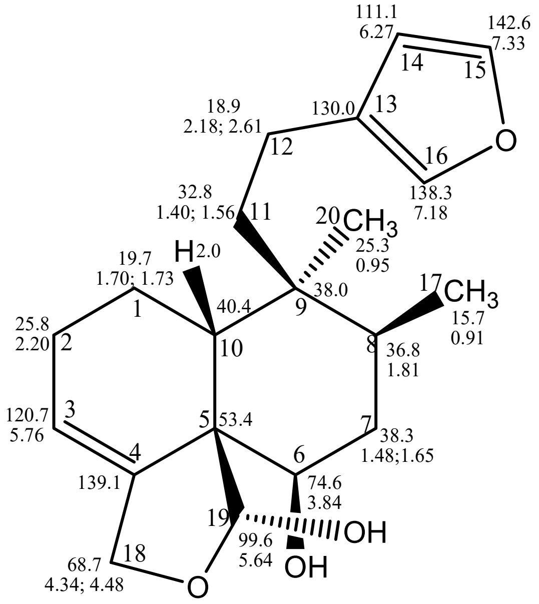

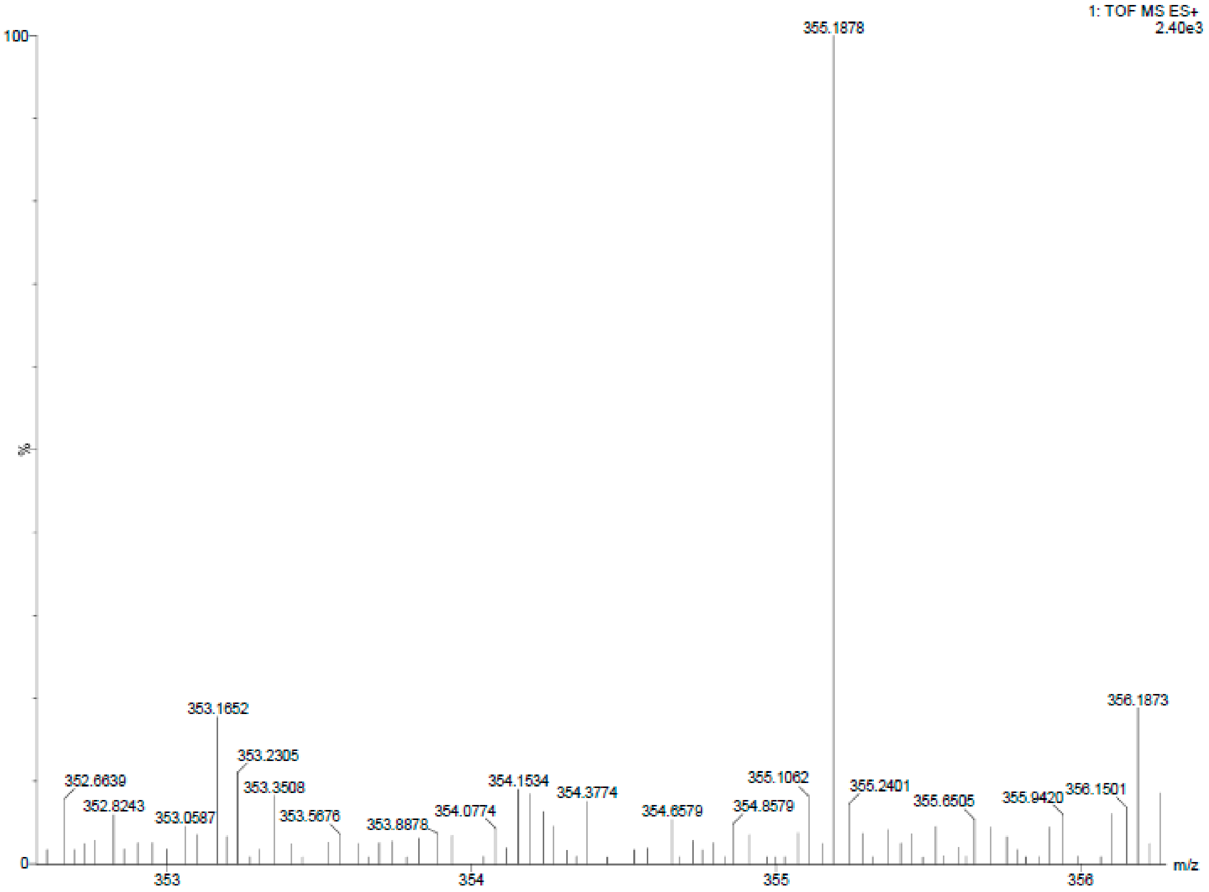

Figure S1: High-resolution mass spectrum of compound **1**.

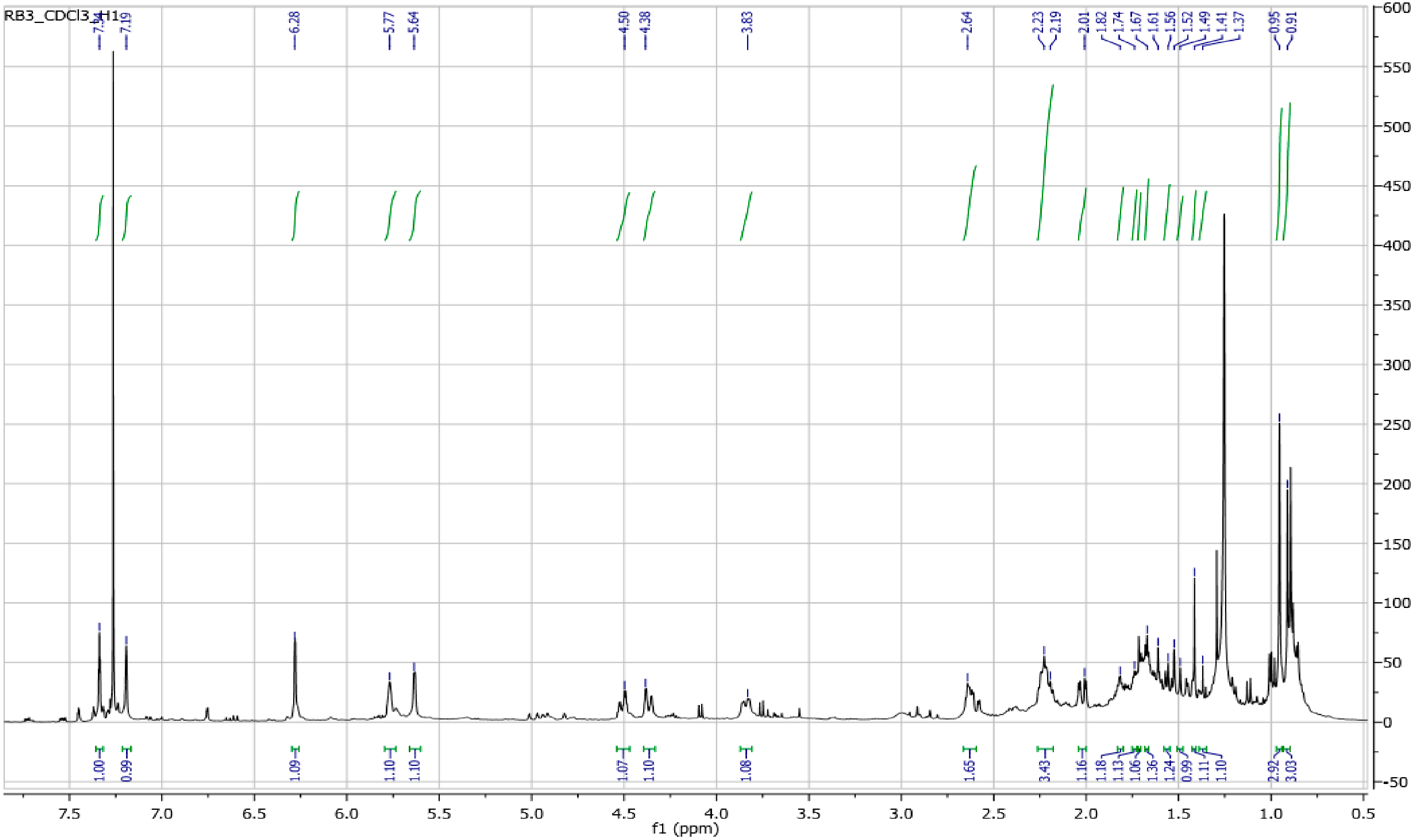

Figure S2: ^{1}H NMR spectrum of compound **1** at 400MHz in $CDCl_3$.

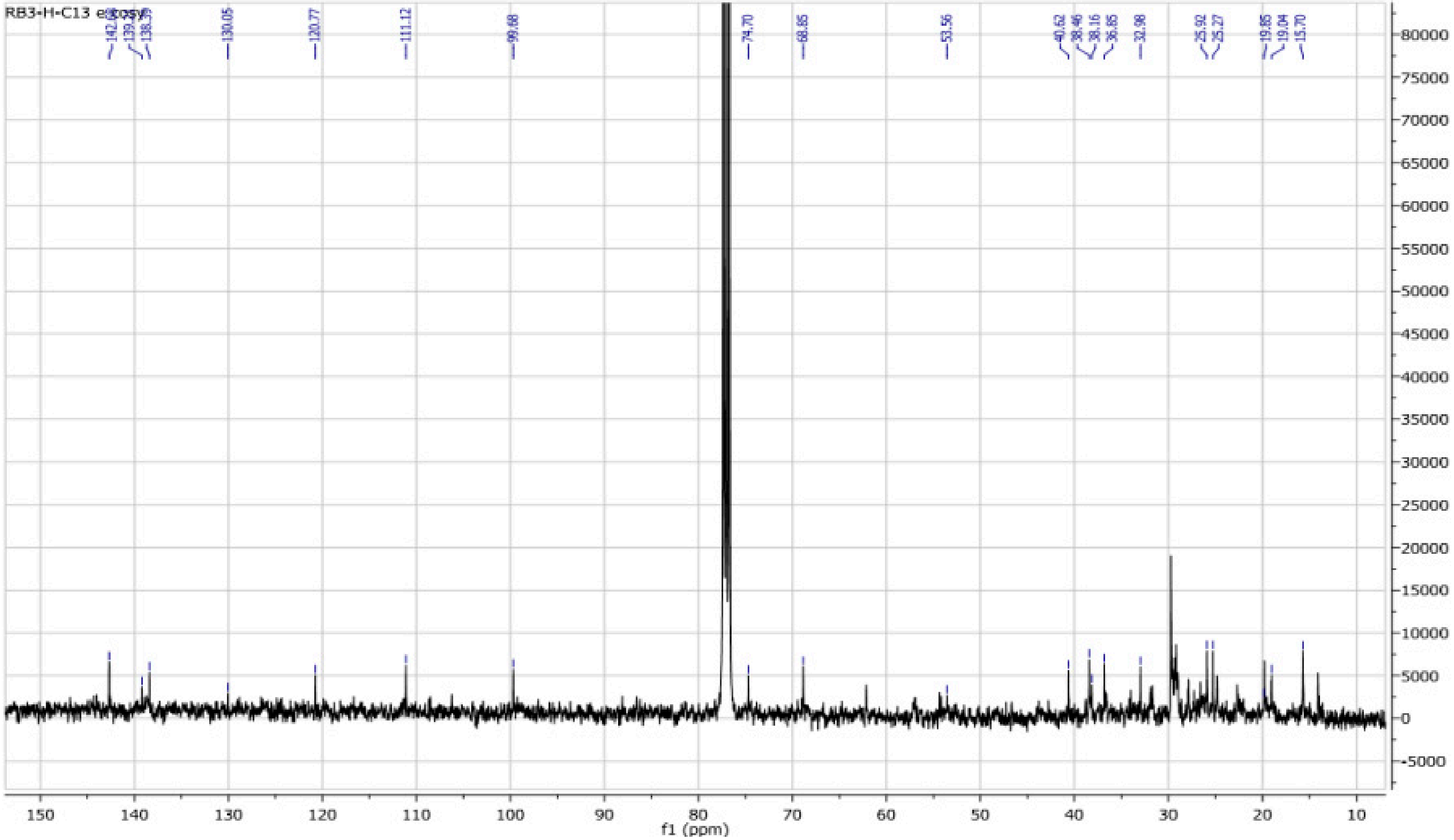

Figure S3: ^{13}C NMR spectrum of compound **1** at 100MHz in $CDCl_3$

b) Compound 2 – $C_{21}H_{30}O_3$

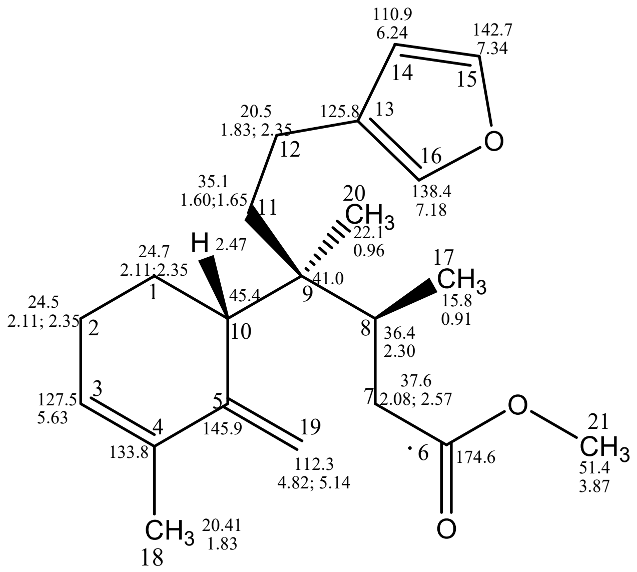

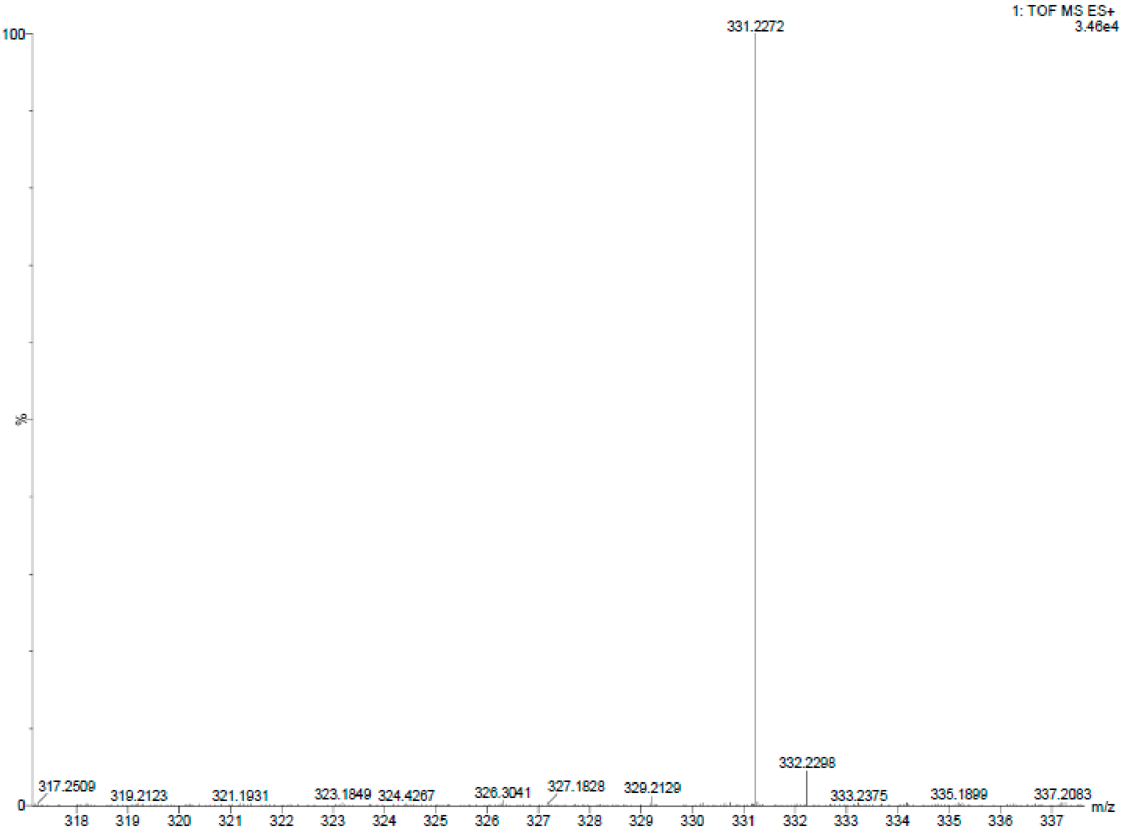

Figure S4: High-resolution mass spectrum of compound **2**.

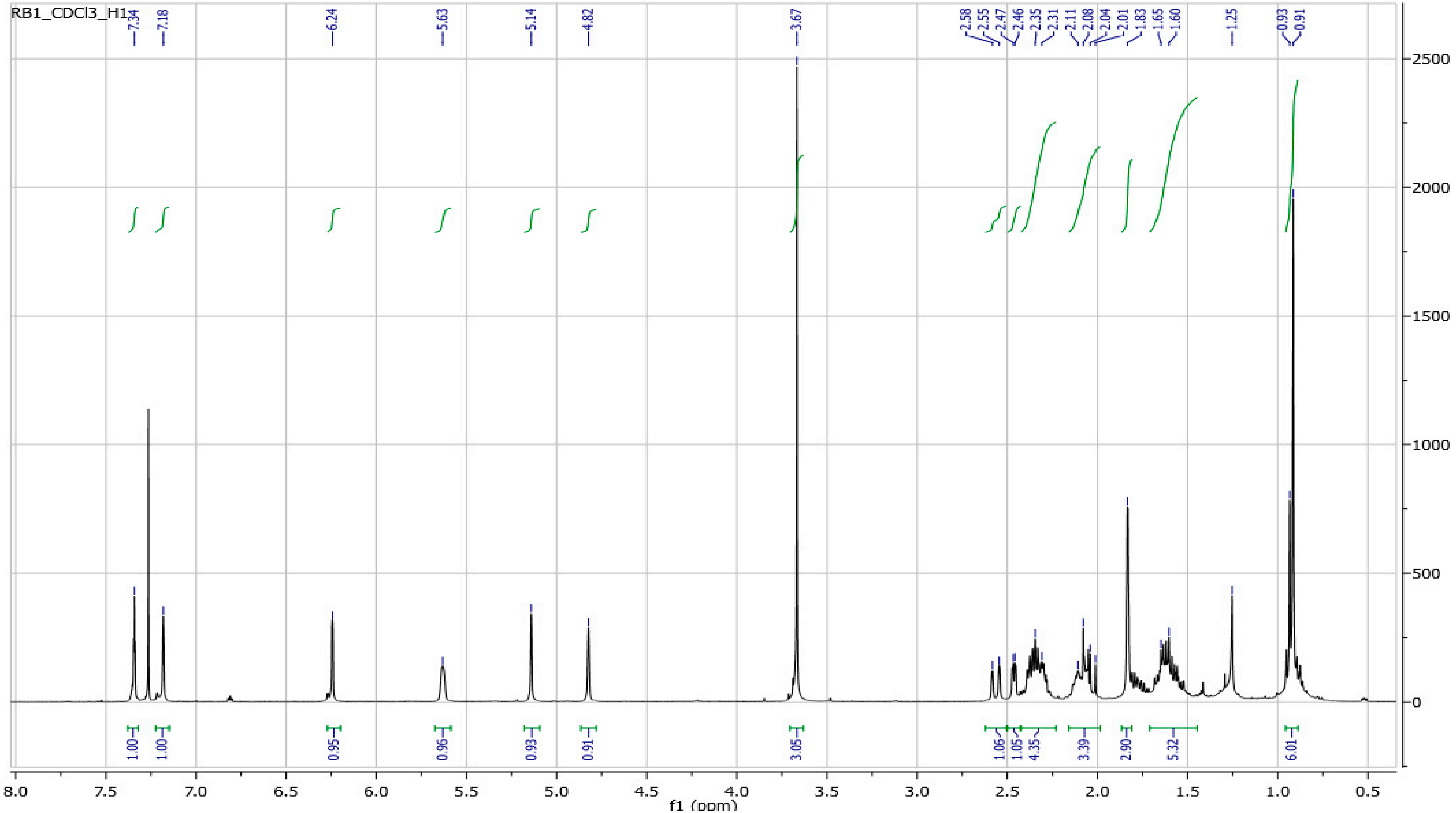

Figure S5: ^{1}H NMR spectrum of compound **2** at 400MHz in $CDCl_3$.

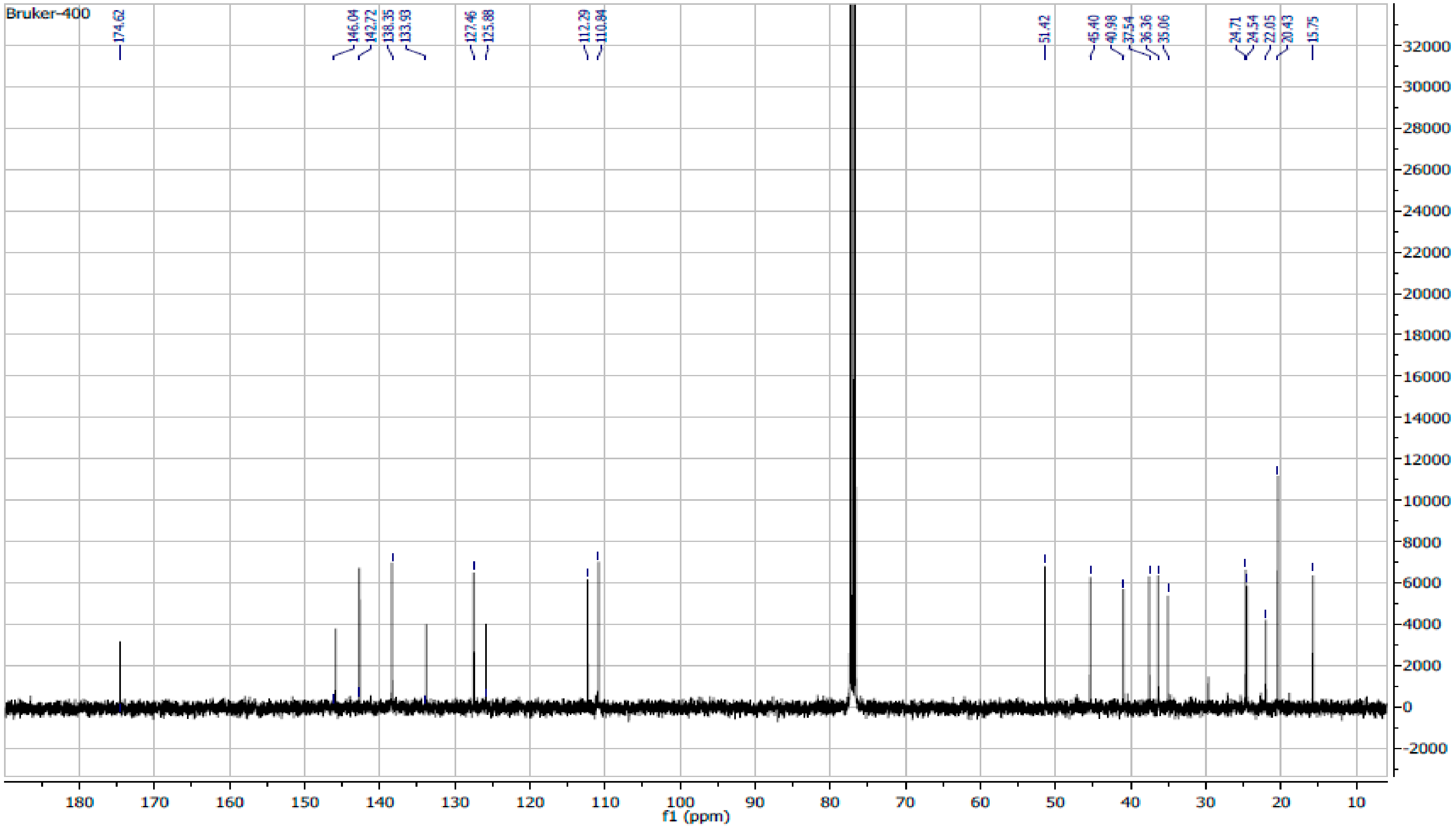

Figure S6: ^{13}C NMR spectrum of compound **2** at 100MHz in $CDCl_3$.

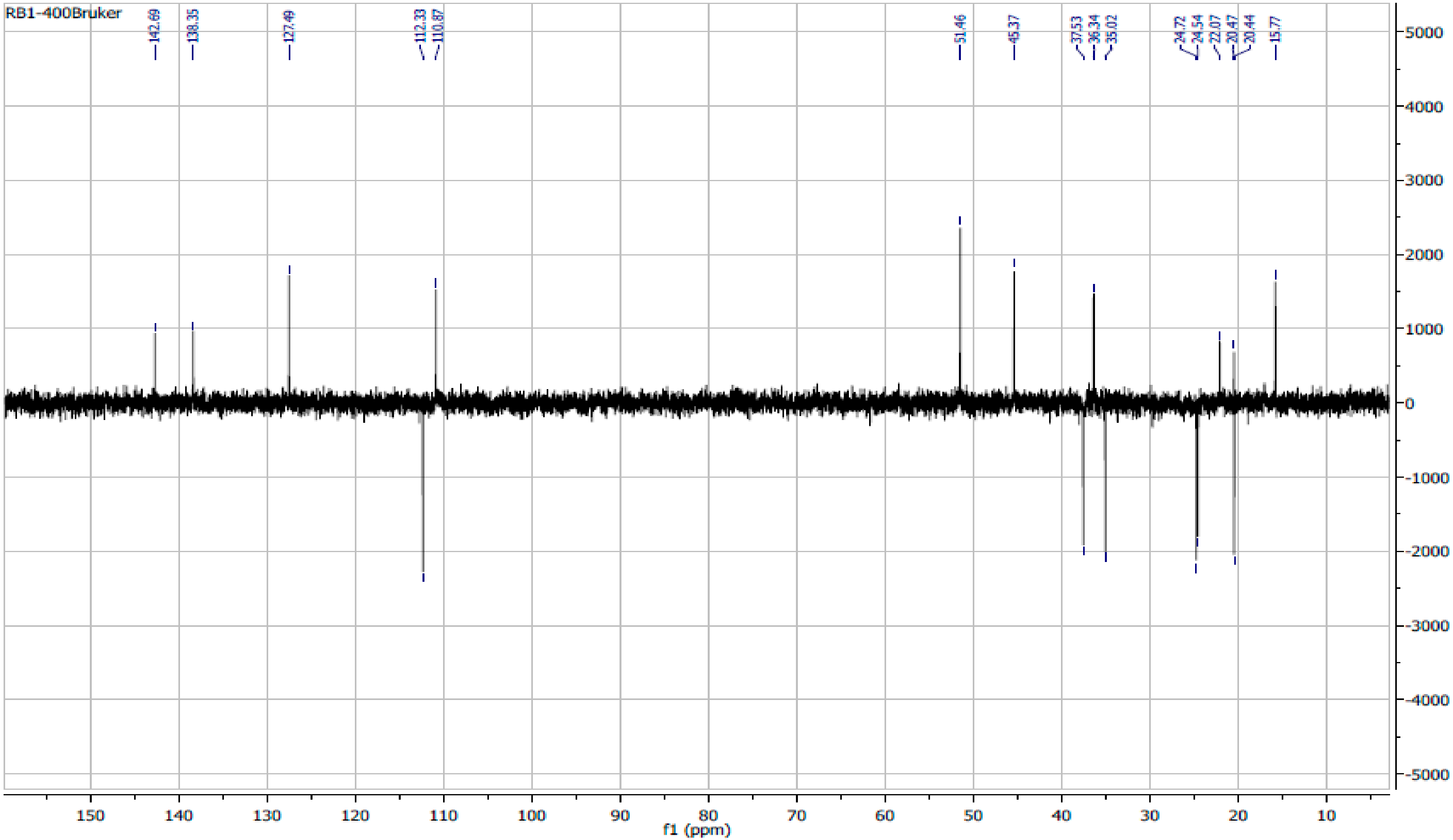

Figure S7: DEPT 135 NMR spectrum of compound **2** at 100MHz in $CDCl_3$.

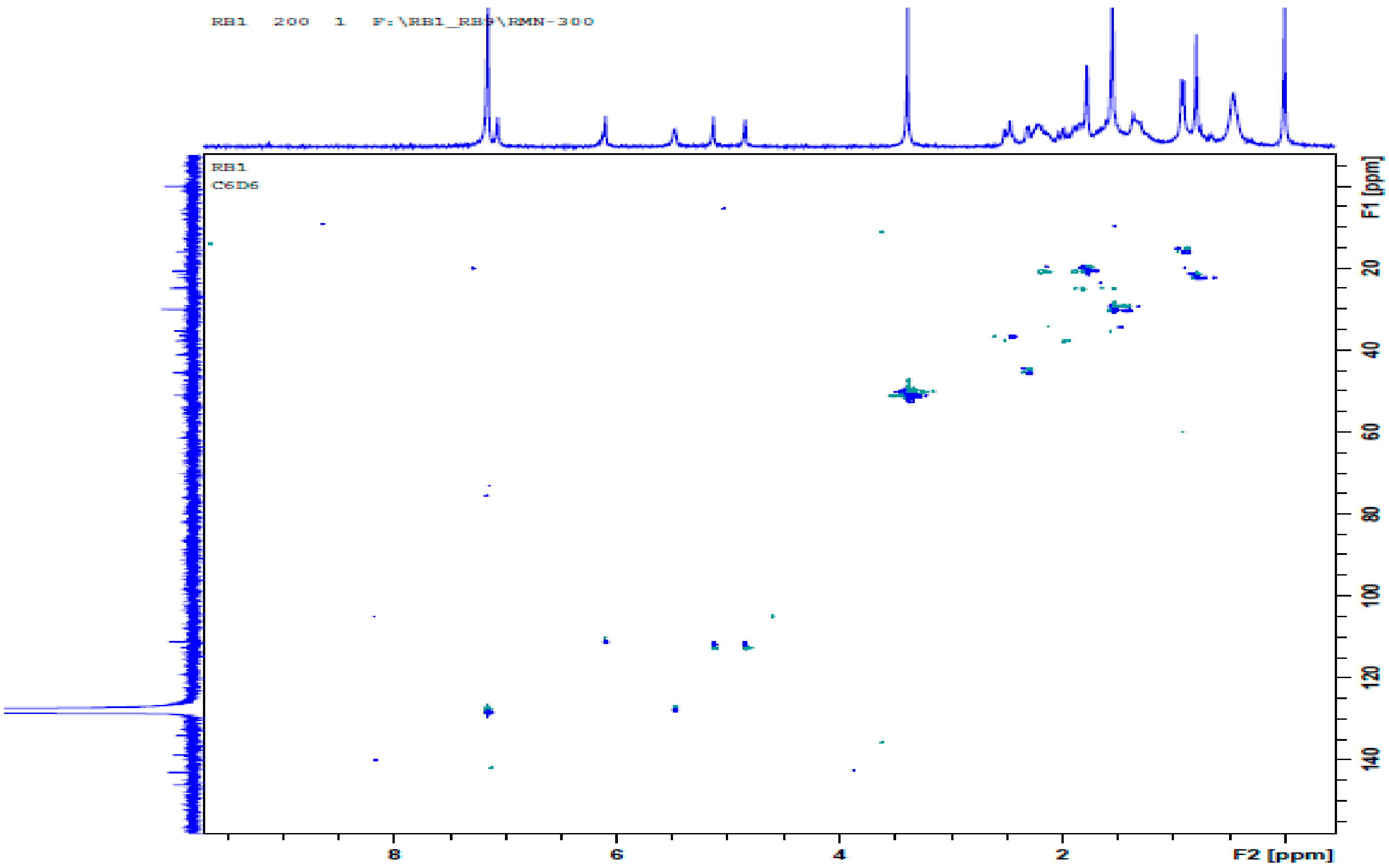

Figure S8: HSQC NMR spectrum of compound **2** at 300MHz in $CDCl_3$.

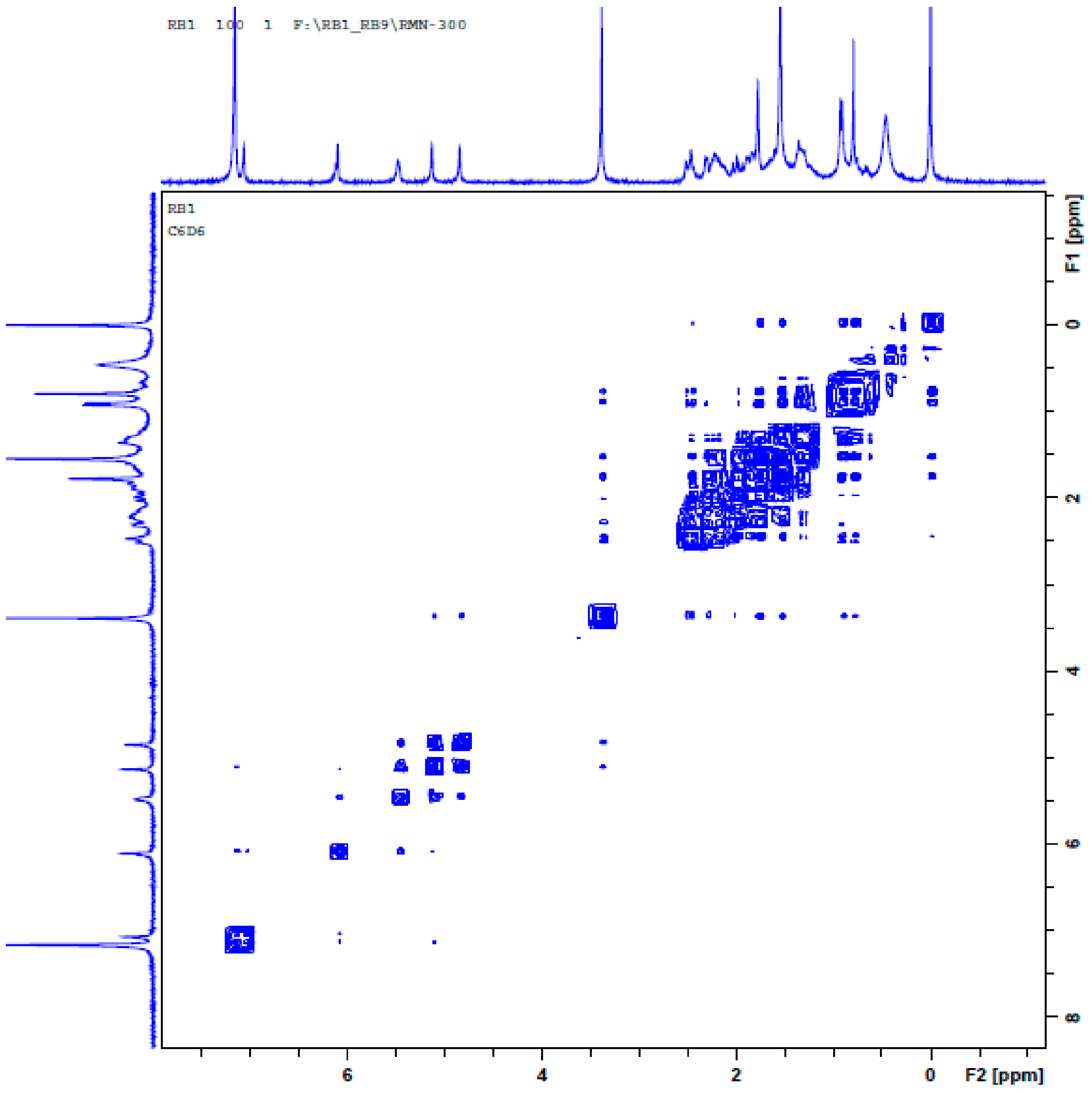

Figure S9: 1H-1H COSY NMR spectrum of compound **2** at 300 MHz in $CDCl_3$.

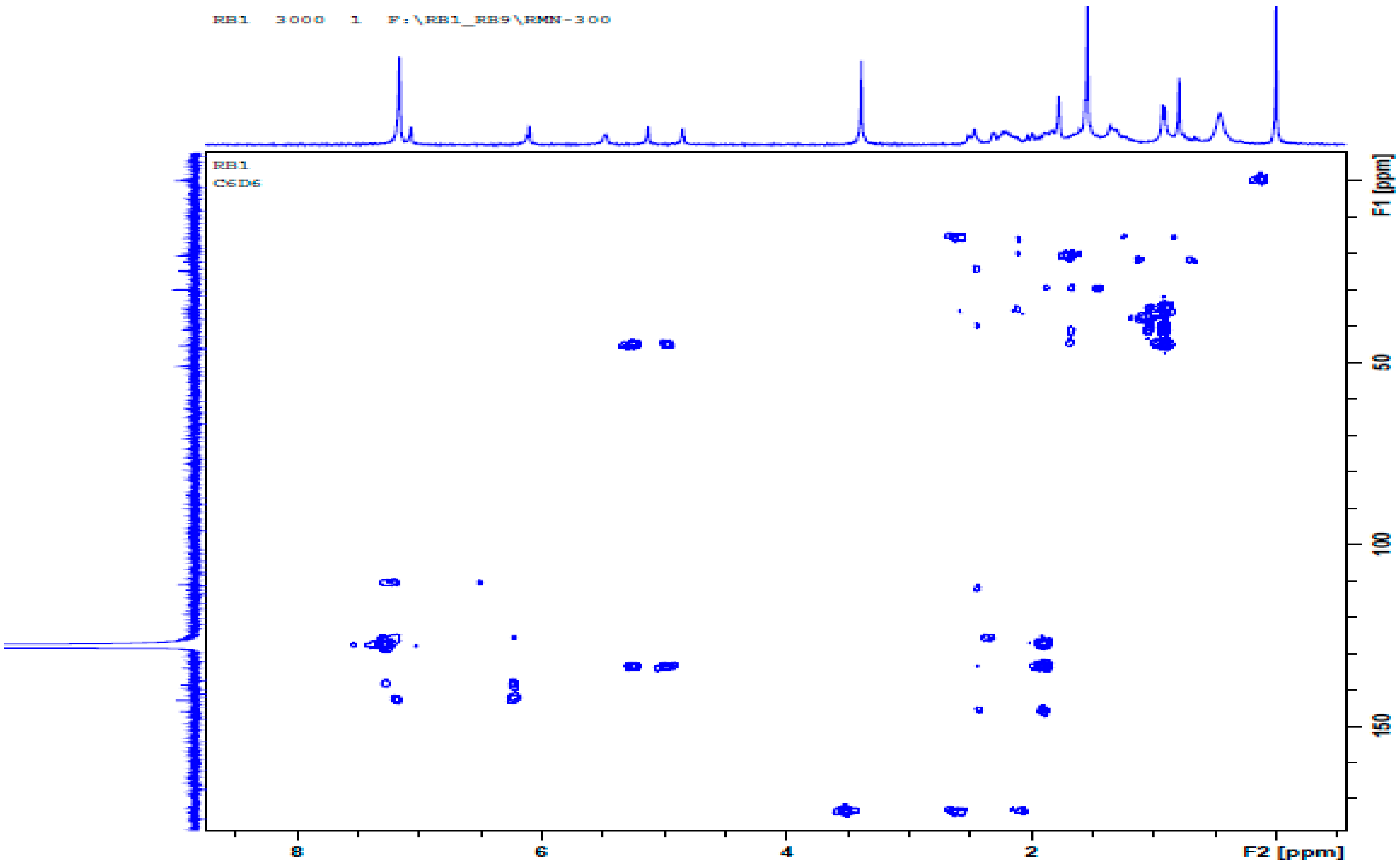

Figure S10: HMBC NMR spectrum of compound **2** at 300 MHz in $CDCl_3$.

c) **Compound 3 – $C_{20}H_{28}O_4$**

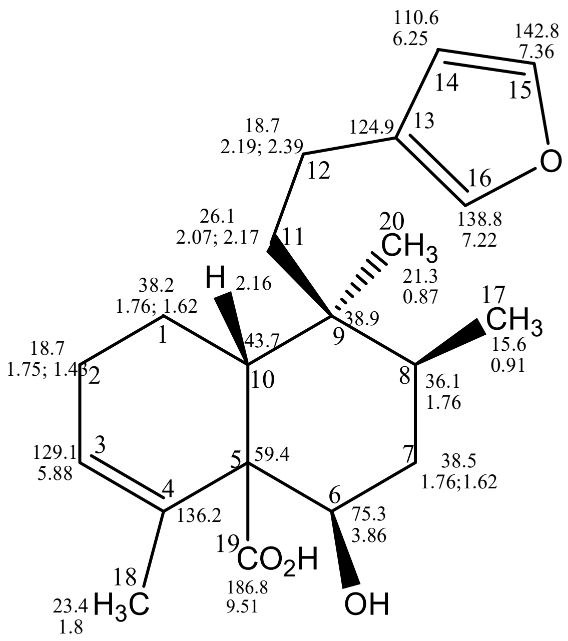

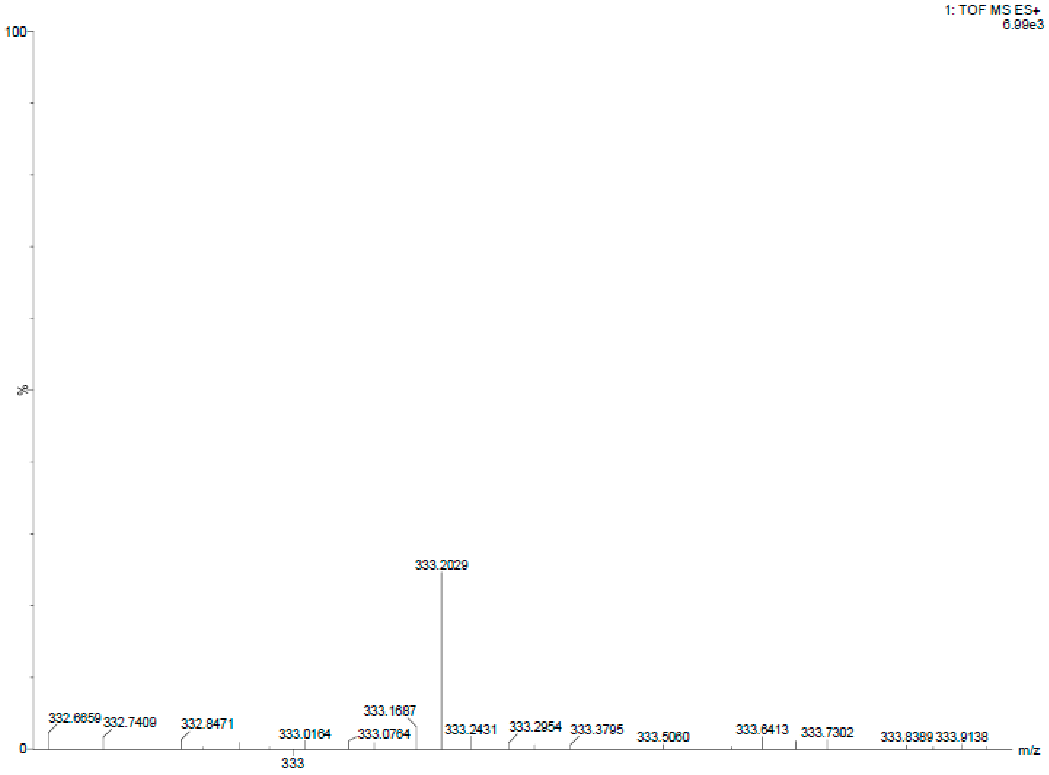

Figure S11 High-resolution mass spectrum of compound **3.**

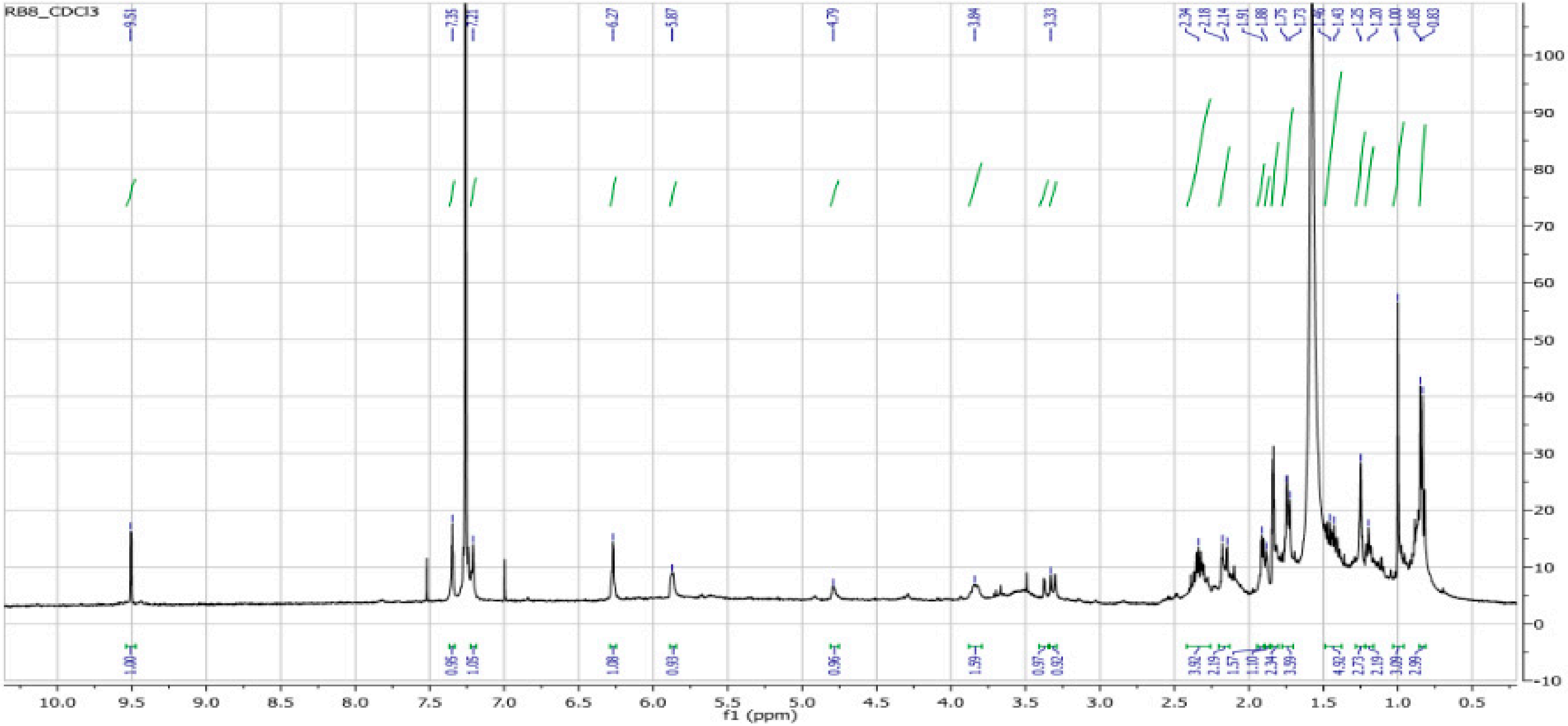

Figure S12: ^{1}H NMR spectrum of compound **3** at 400MHz in $CDCl_3$.

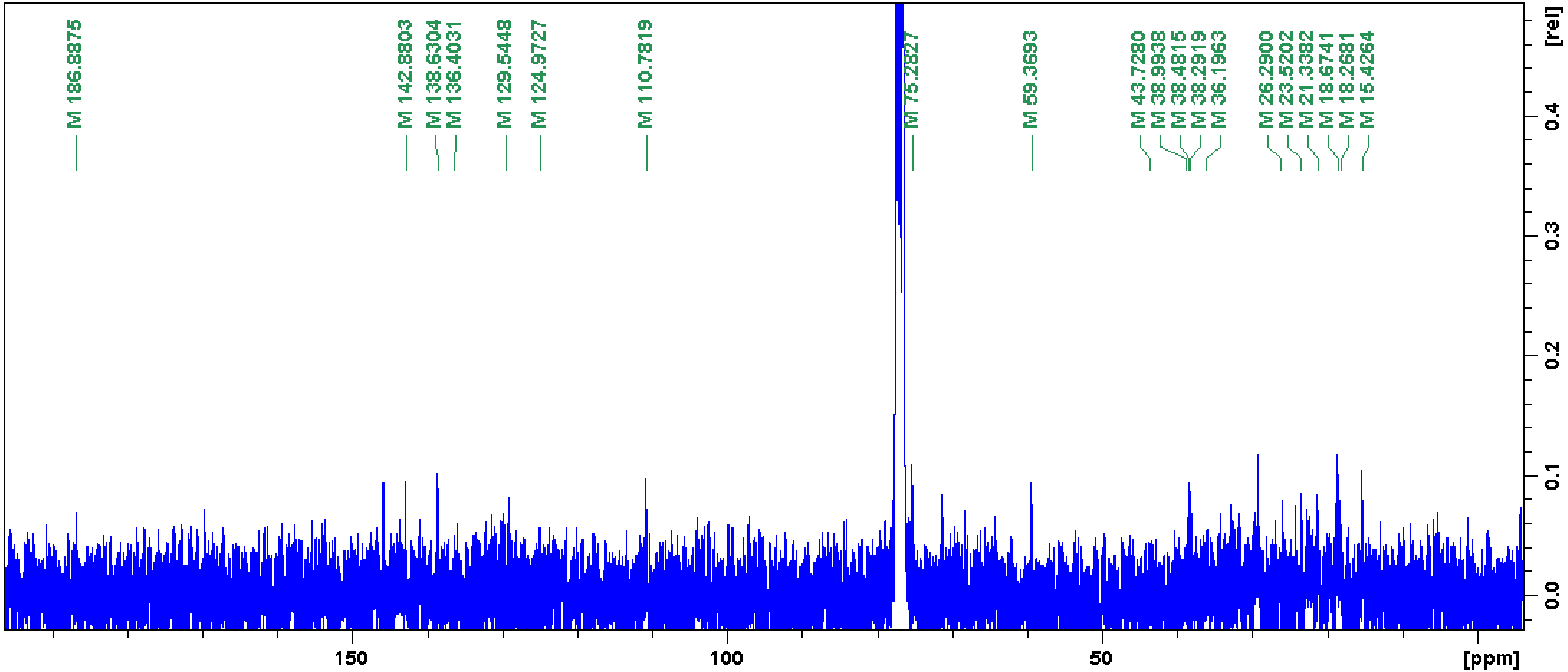

Figure S13: ^{13}C NMR spectrum of compound **3** at 75MHz in $CDCl_3$.

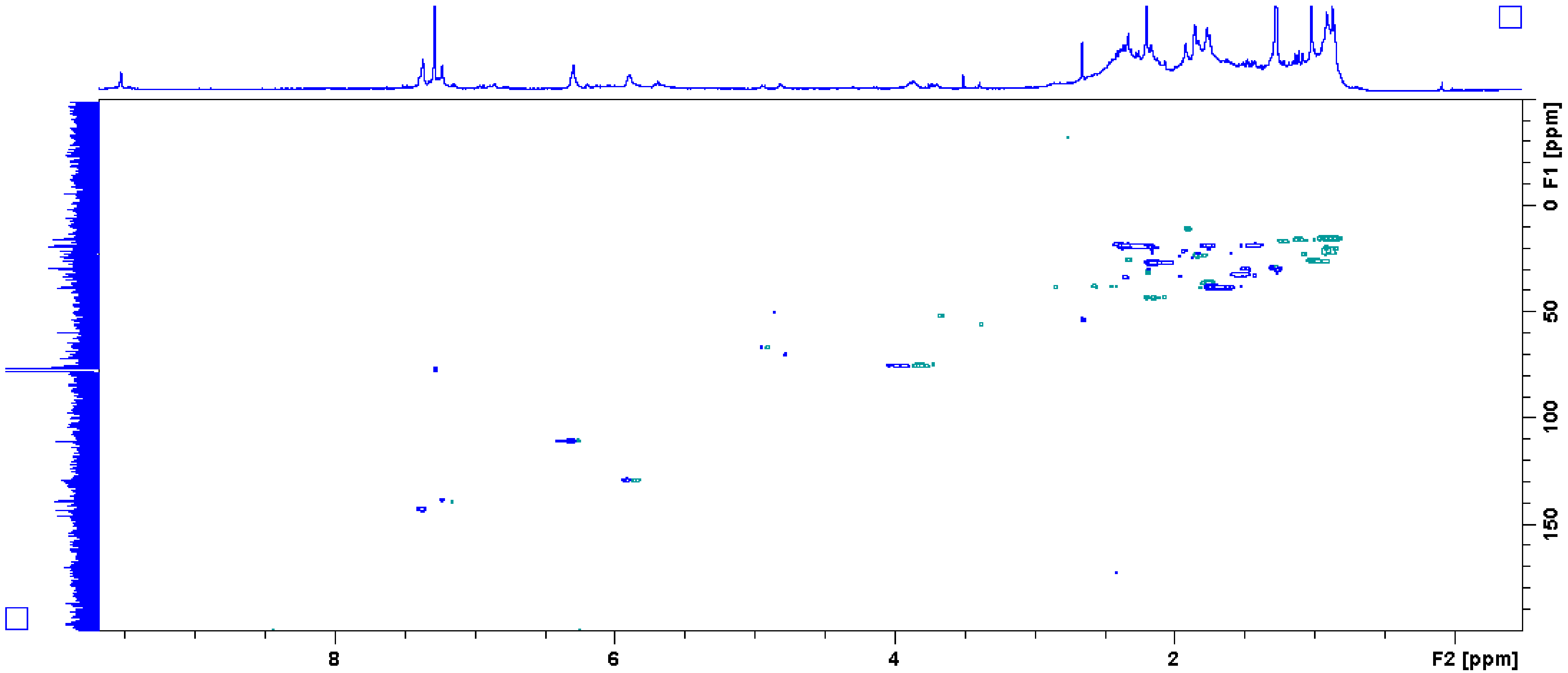

Figure S14: .HSQC NMR spectrum of compound **3** at 300MHz in $CDCl_3$.

d) Compound 4 - $C_{21}H_{30}O_4$

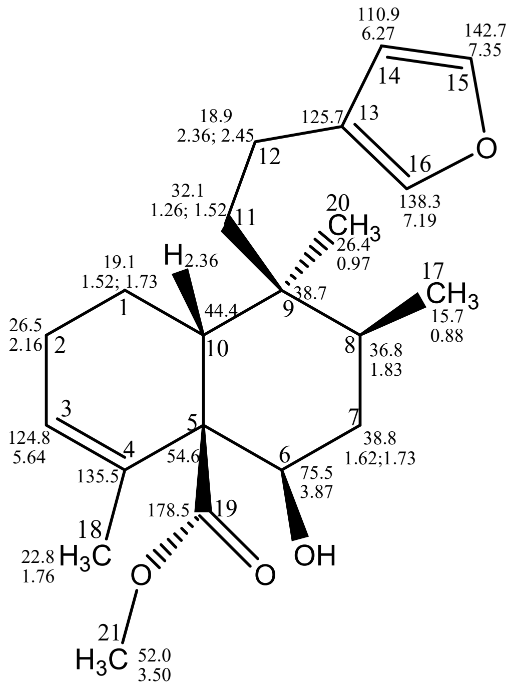

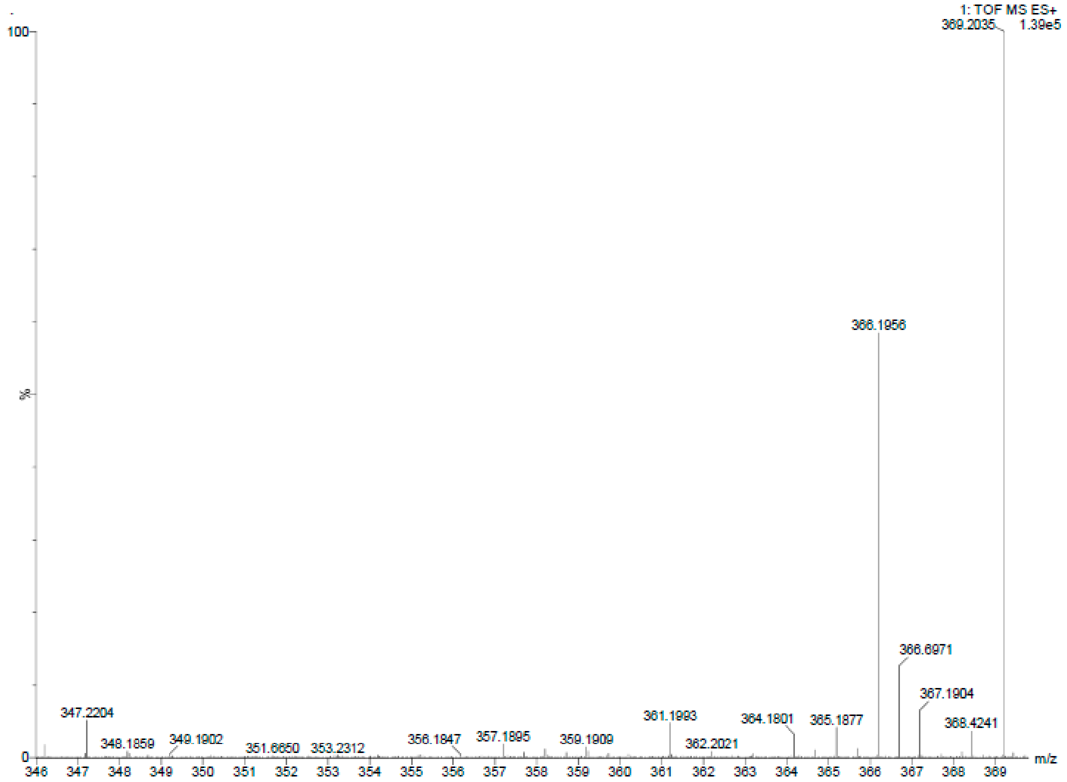

Figure S15: High-resolution mass spectrum of compound **3.**

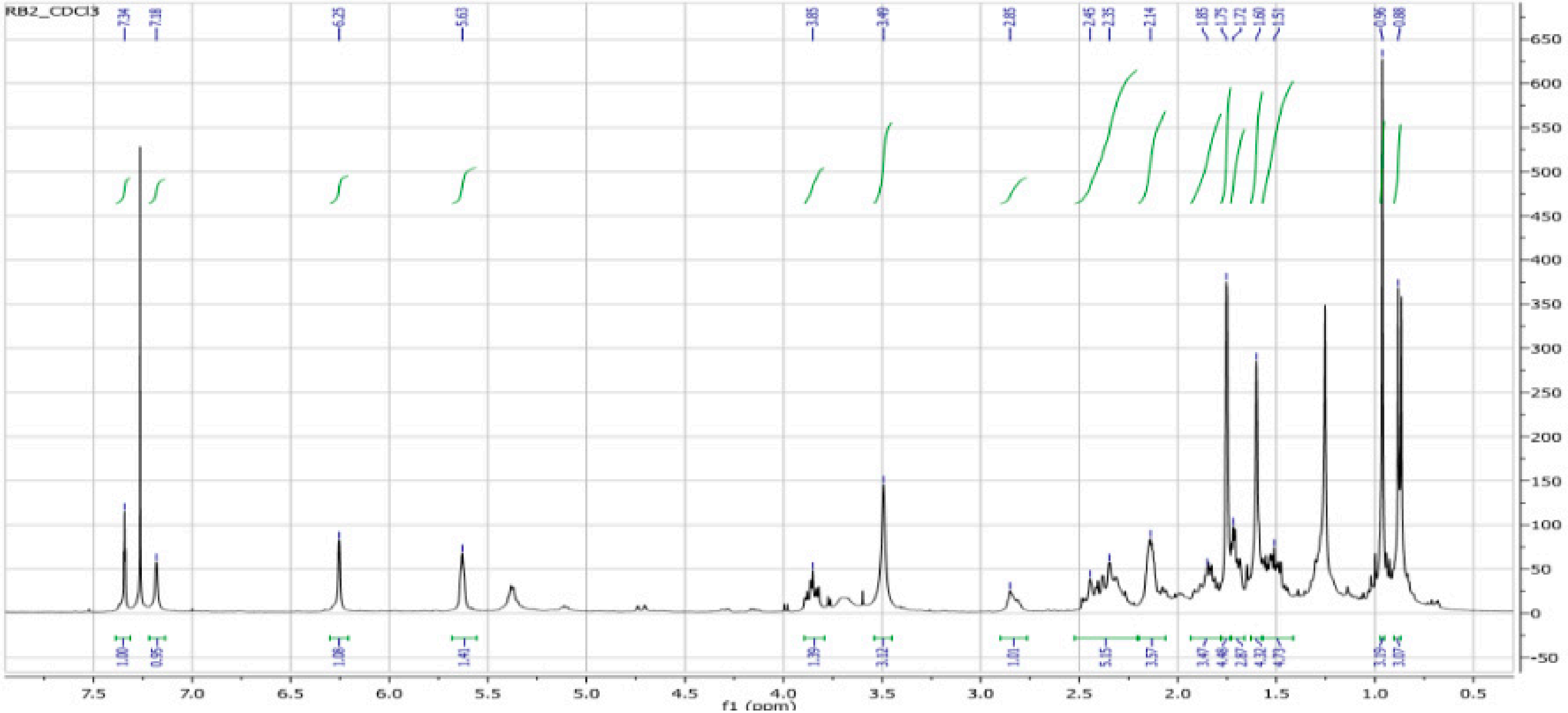

Figure S16: ^{1}H NMR spectrum of compound **4** at 400MHz in $CDCl_3$.

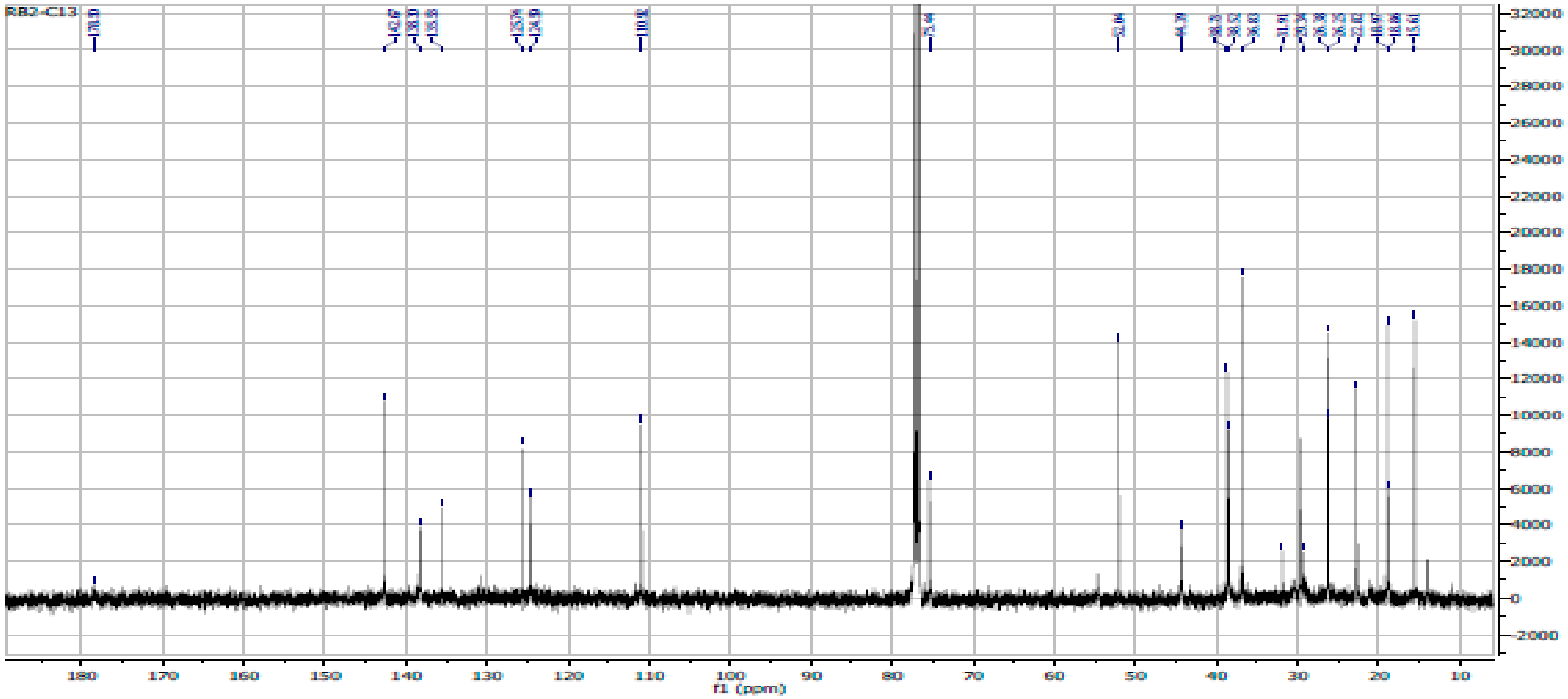

Figure S17: ^{13}C NMR spectrum of compound **4** at 100MHz in $CDCl_3$.

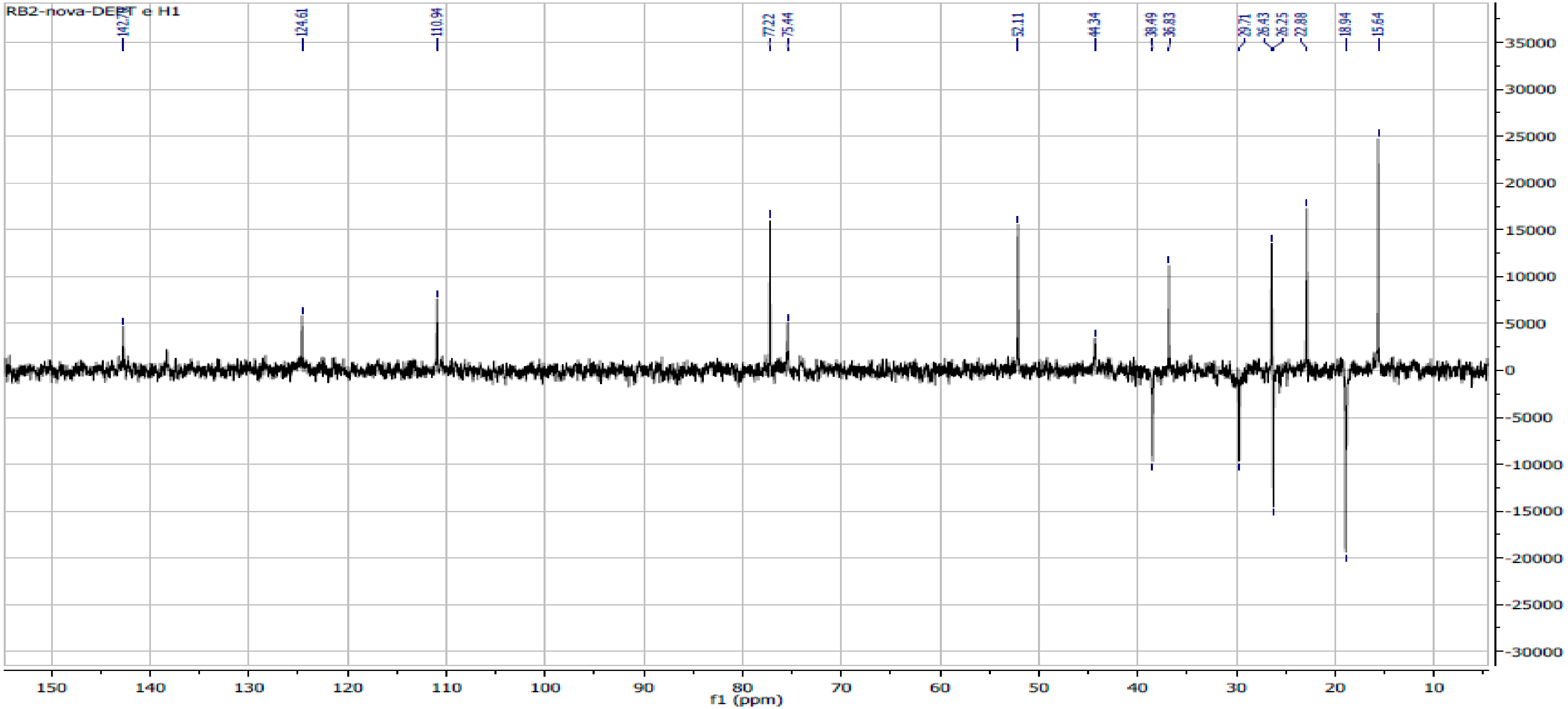

Figure S18: DEPT 135 NMR spectrum of compound 4 at 100MHz in $CDCl_3$.

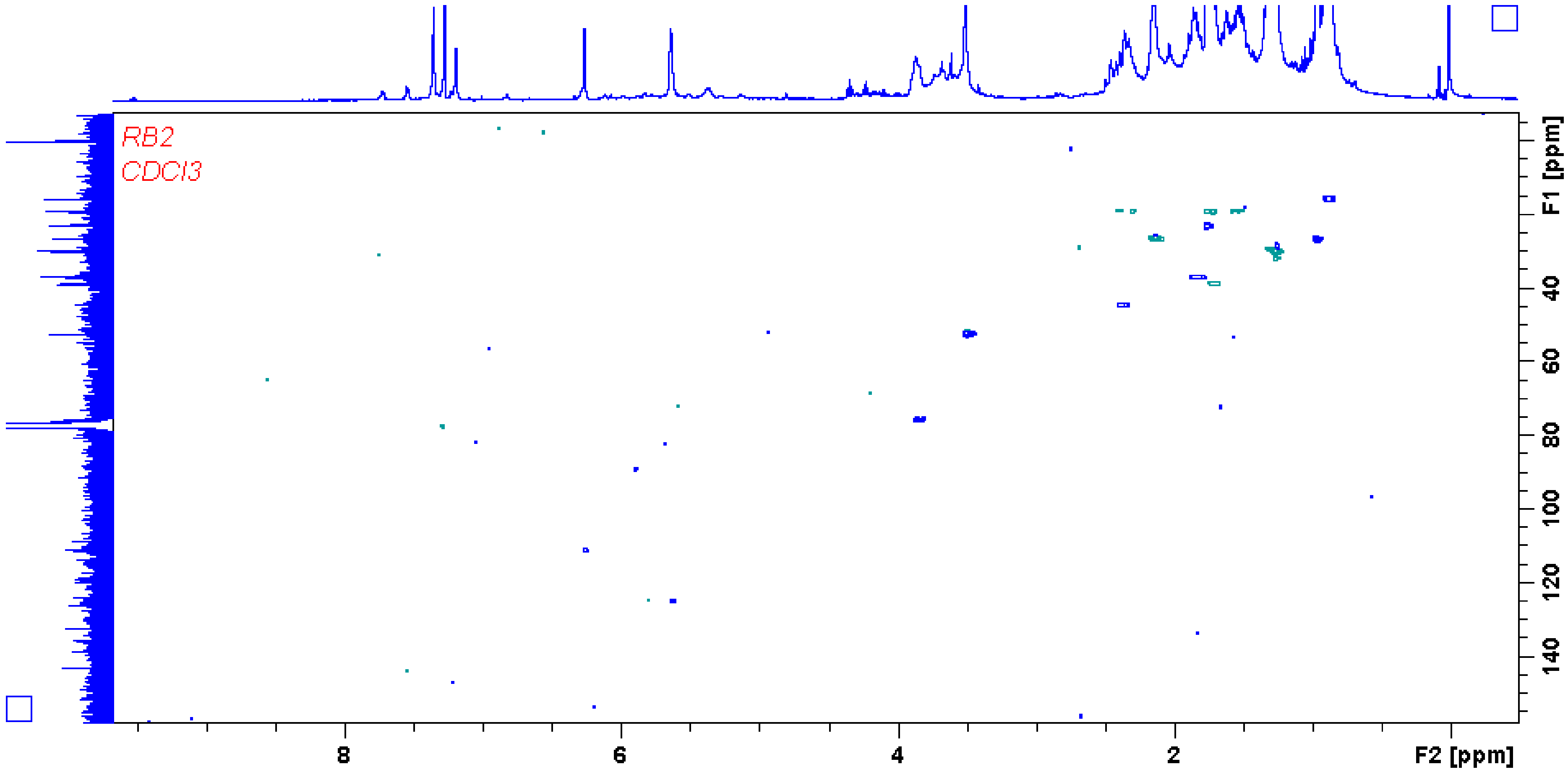

Figure S19: .HSQC NMR spectrum of compound **4** at 300MHz in $CDCl_3$.

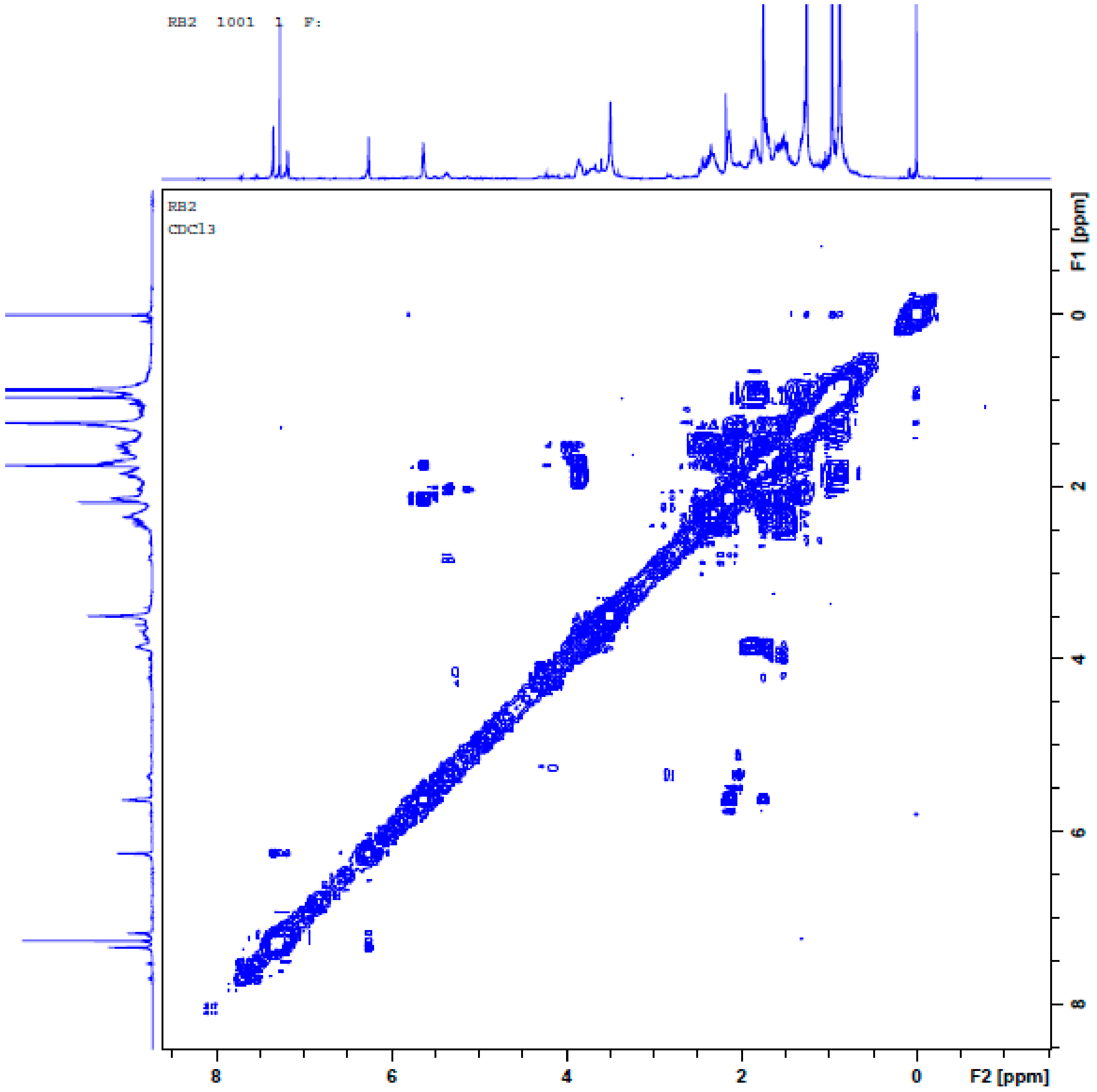

Figure S20: 1H-1H COSY NMR spectrum of compound **4** at 300 MHz in $CDCl_3$.

.

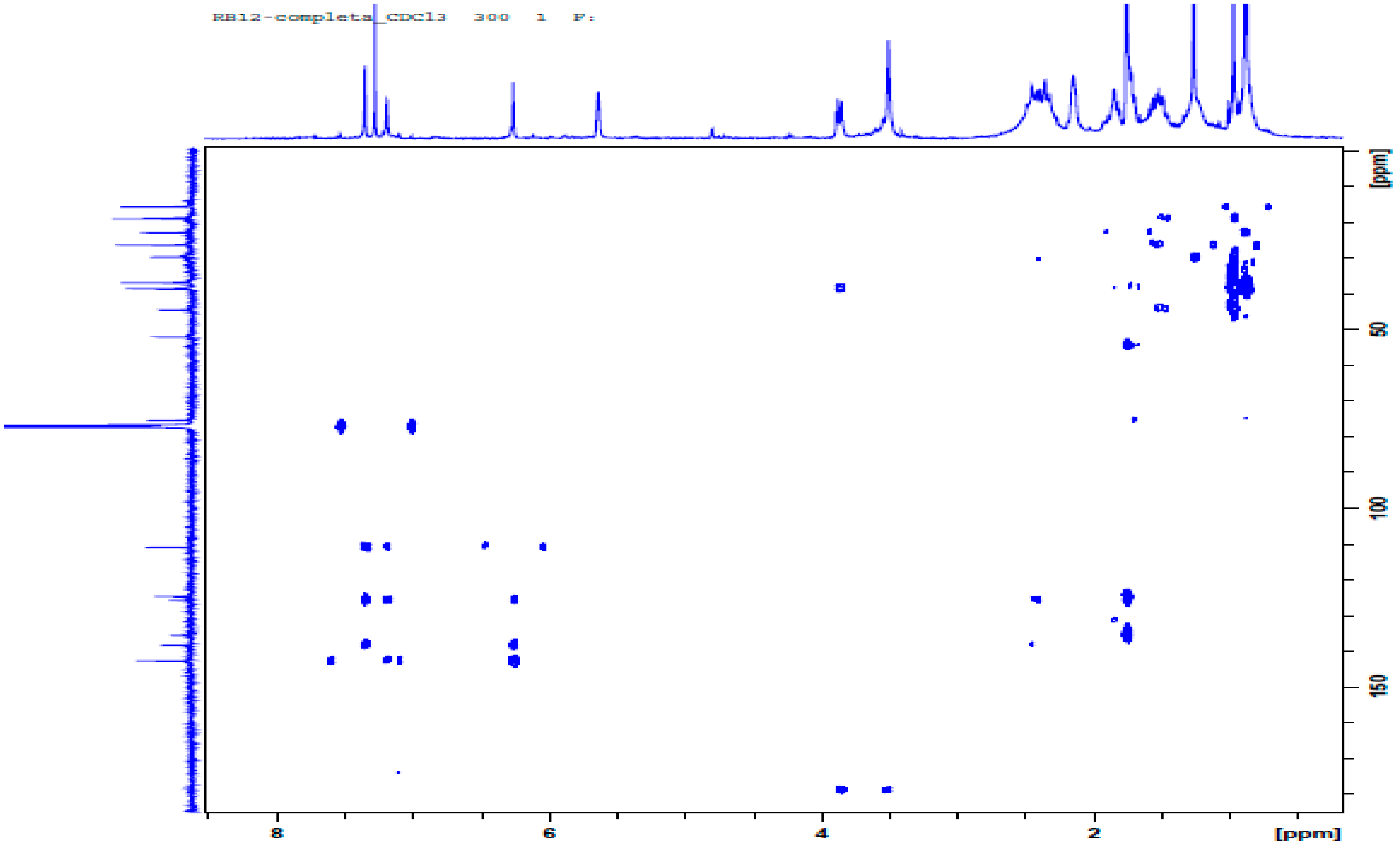

Figure S21: HMBC NMR spectrum of compound **4** at 300 MHz in $CDCl_3$

e) **Compound 5 – $C_{20}H_{30}O$**

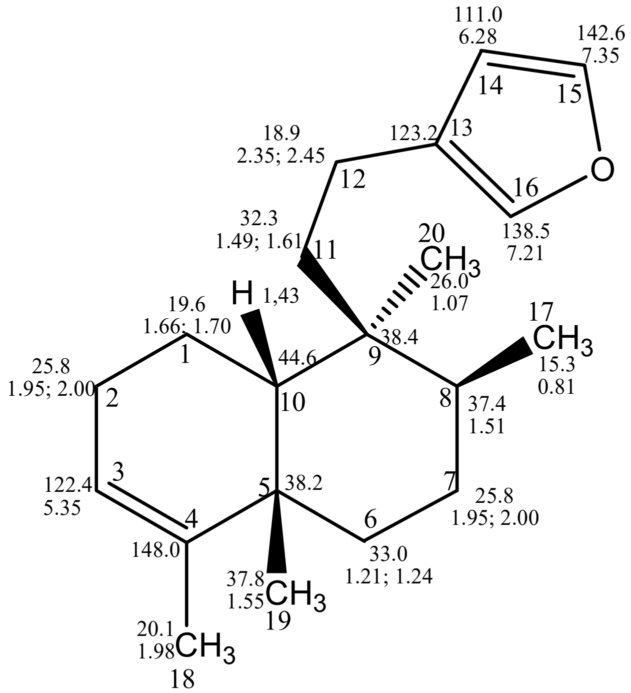

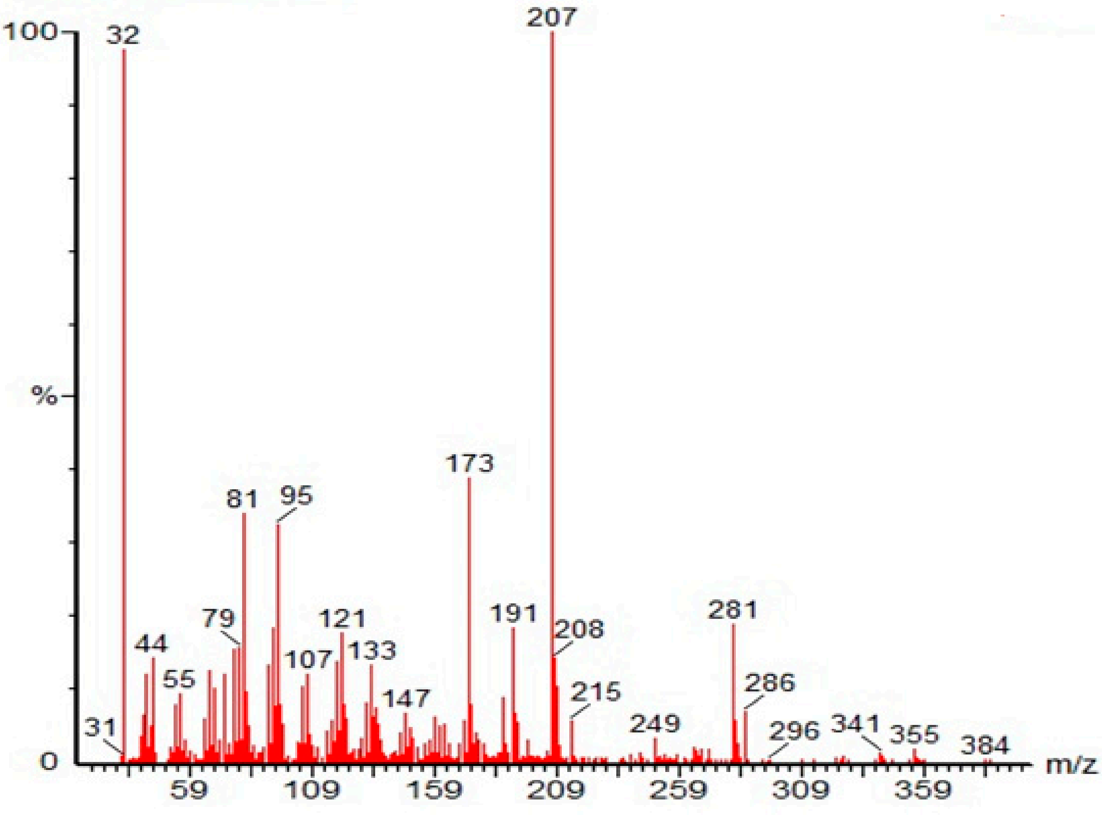

Figure S22: (+)-Electron Impact Mass Spectrum of compound **5**

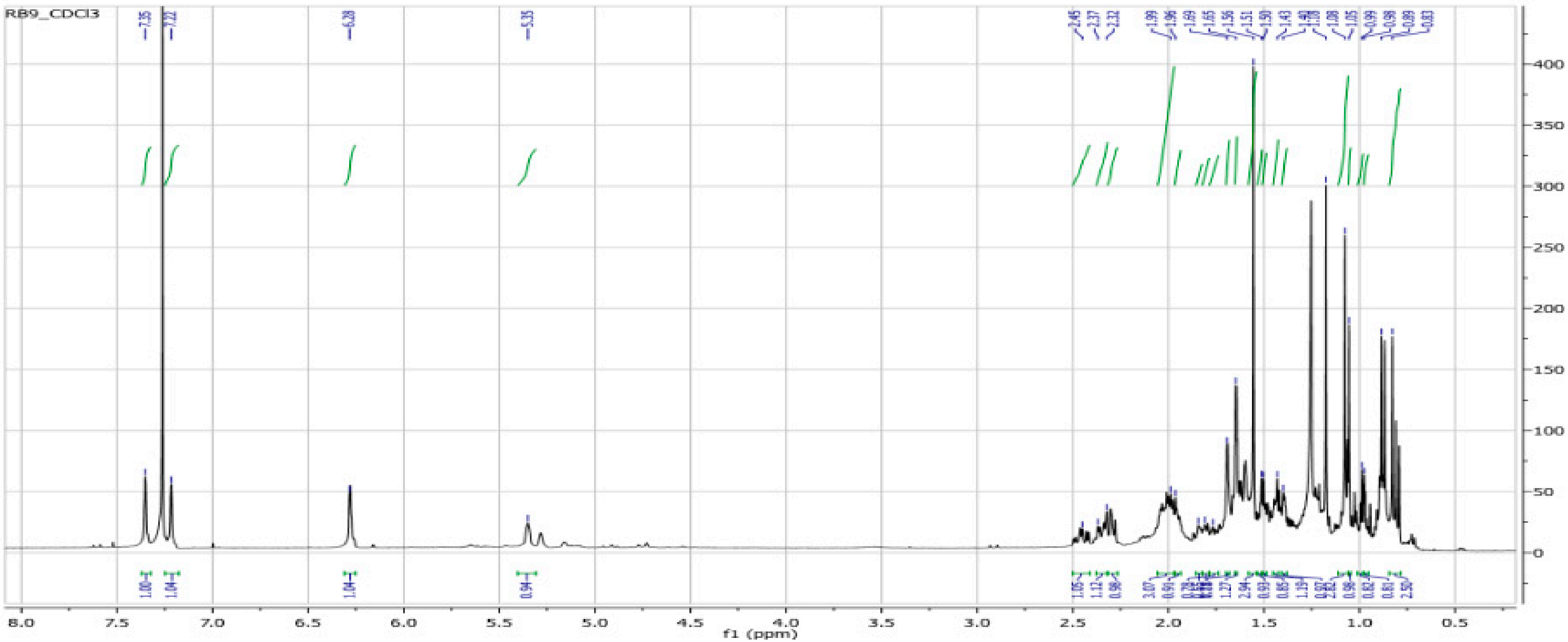

Figure S23: ^{1}H NMR spectrum of compound **5** at 400MHz in $CDCl_3$.

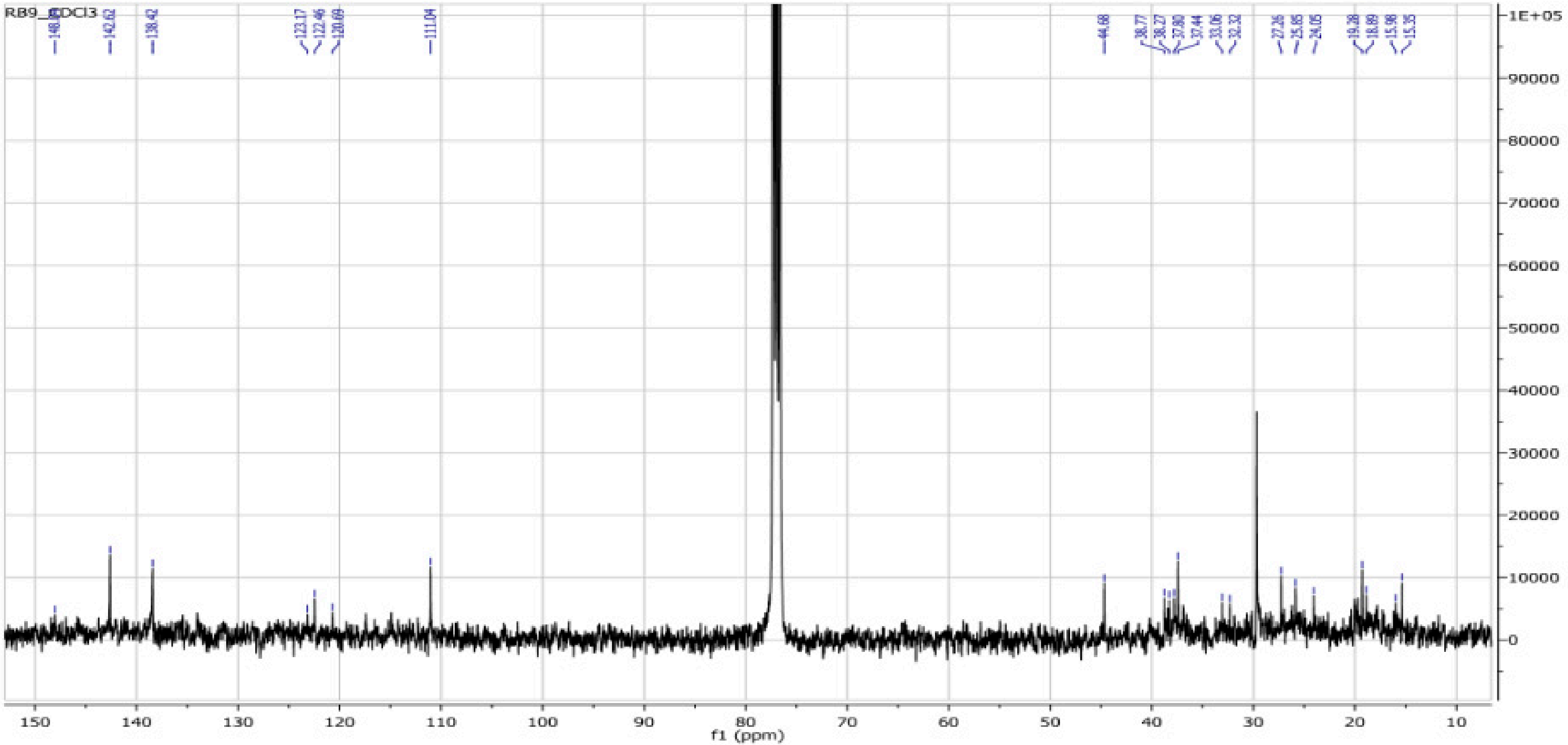

Figure S24: ^{13}C NMR spectrum of compound **5** at 100 MHz in $CDCl_3$

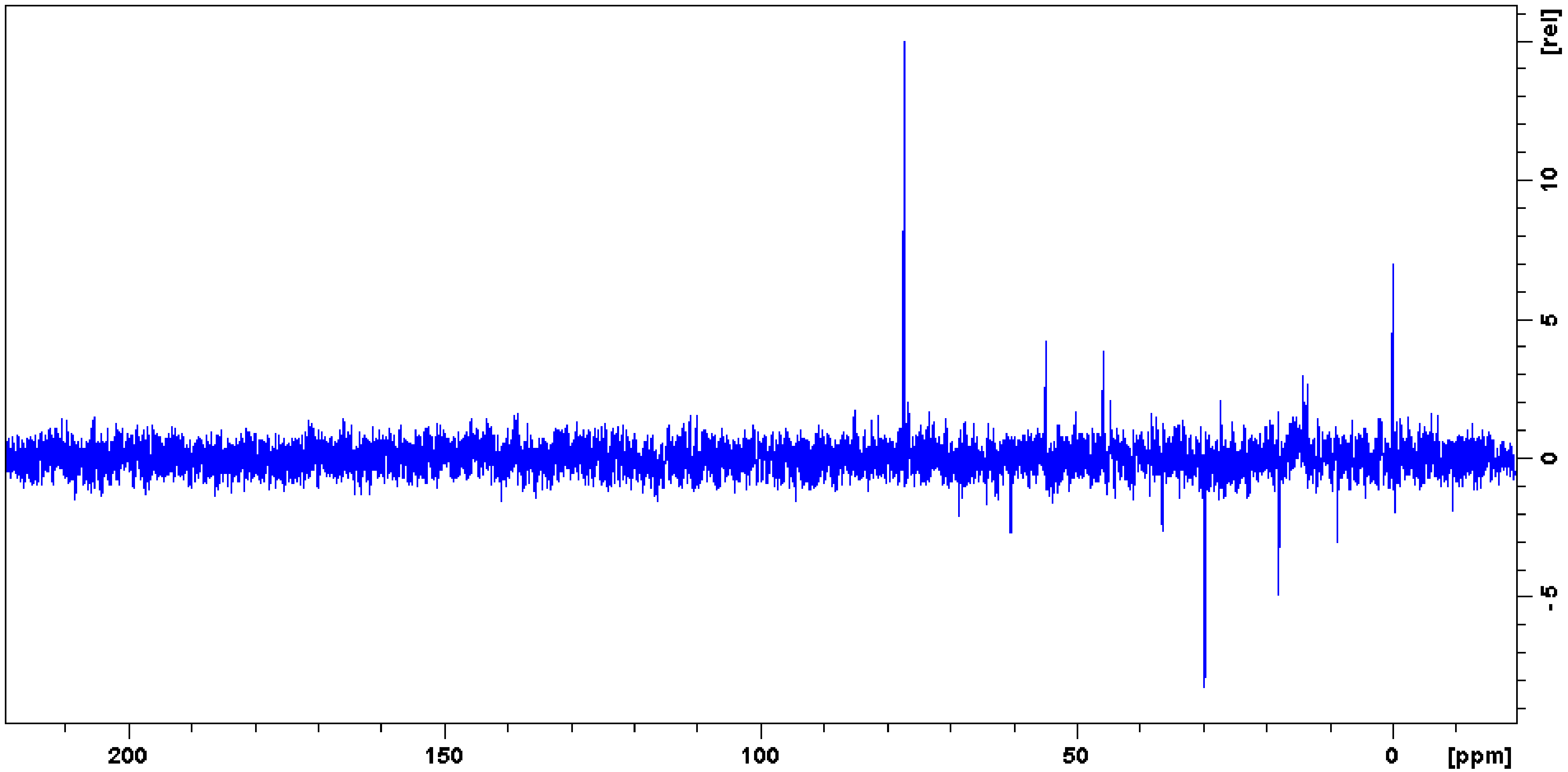

Figure S25: DEPT 135 NMR spectrum of compound **5** at 100MHz in $CDCl_3$.

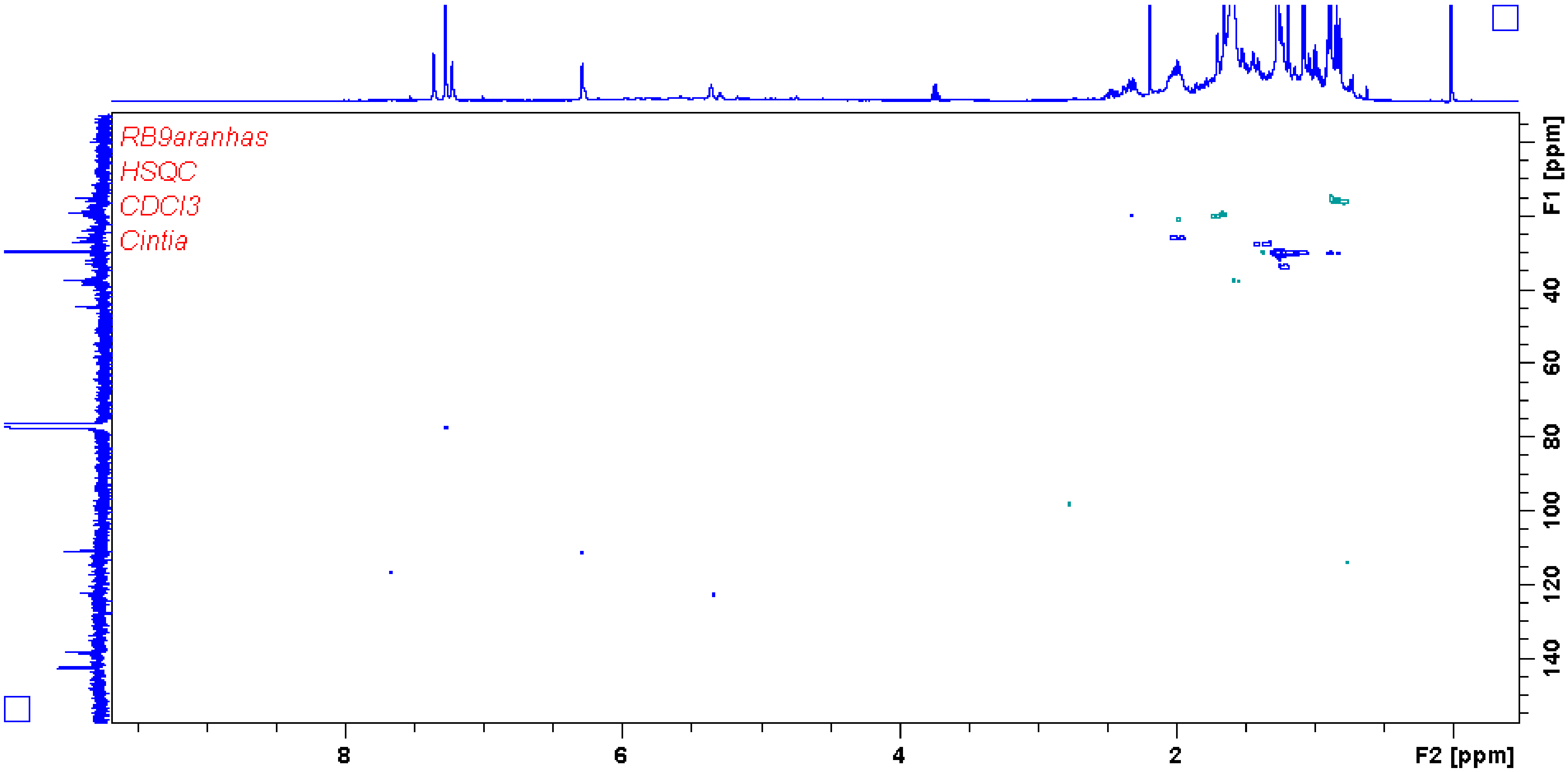

Figure S26: .HSQC NMR spectrum of compound **5** at 300MHz in $CDCl_3$.

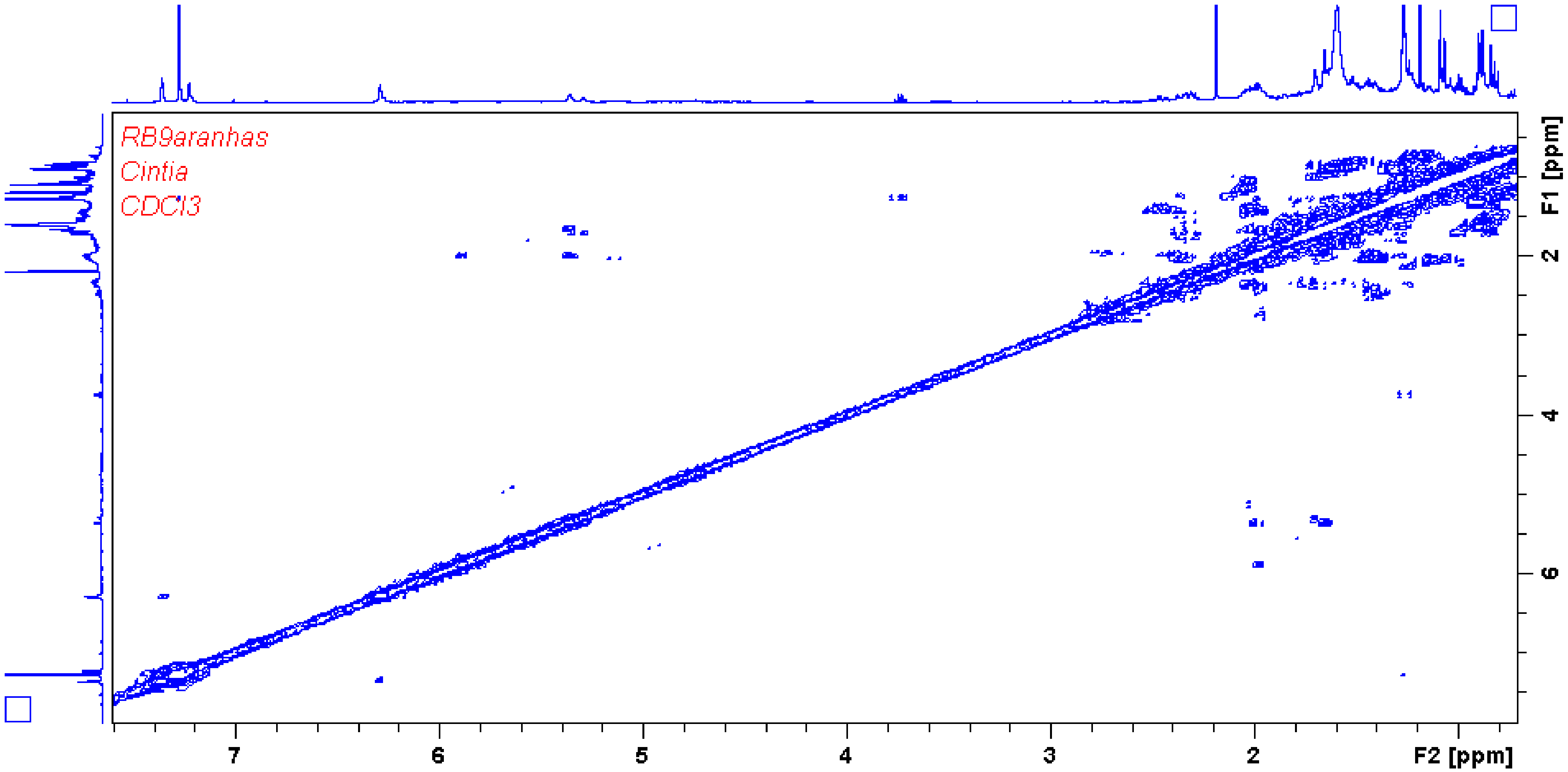

Figure S27: 1H-1H COSY NMR spectrum of compound 5 at 300 MHz in $CDCl_3$.

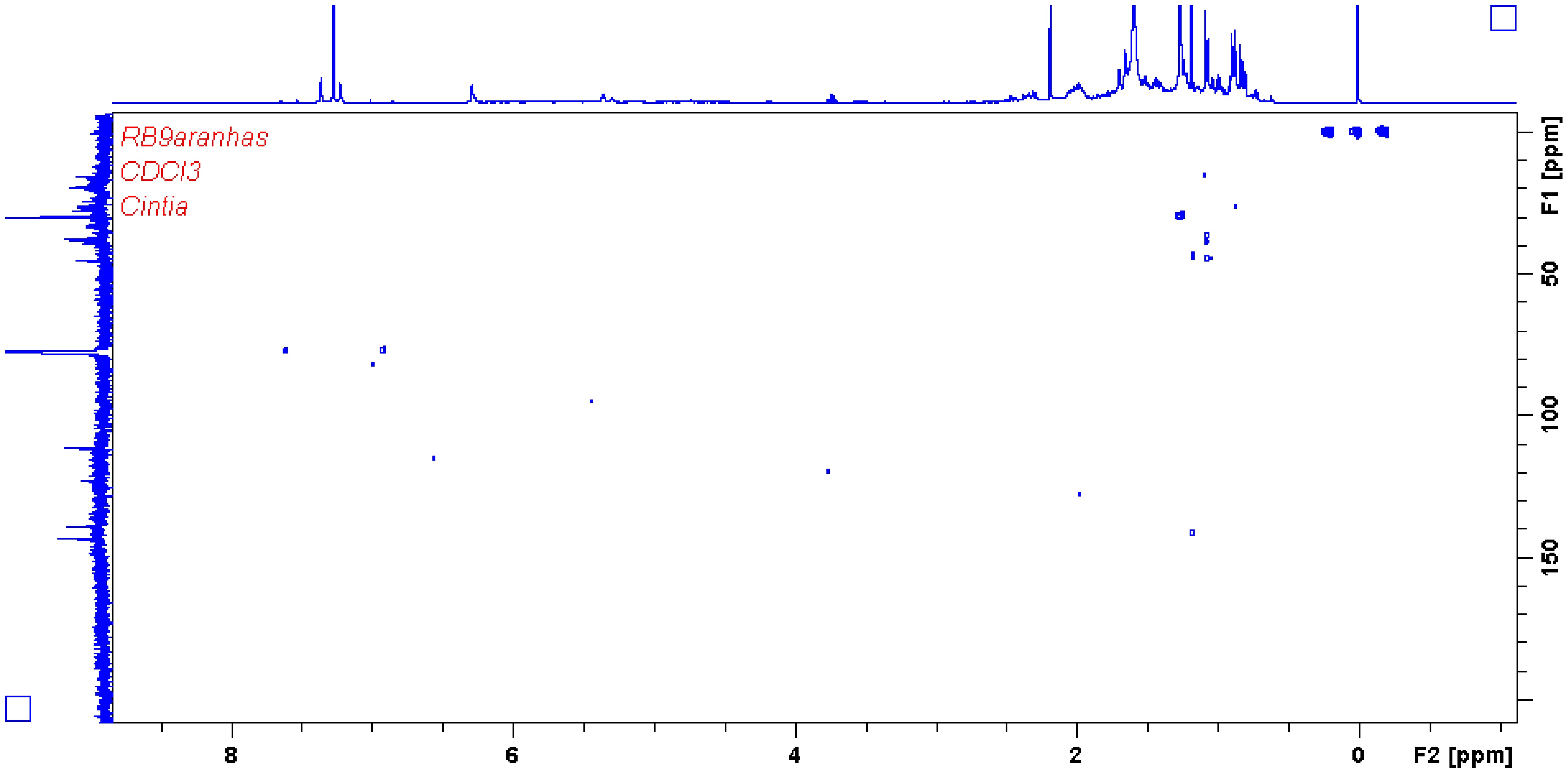

Figure S28: HMBC NMR spectrum of compound 5 at 300 MHz in $CDCl_3$.

e) Compound 6 – $C_{20}H_{30}O_2$

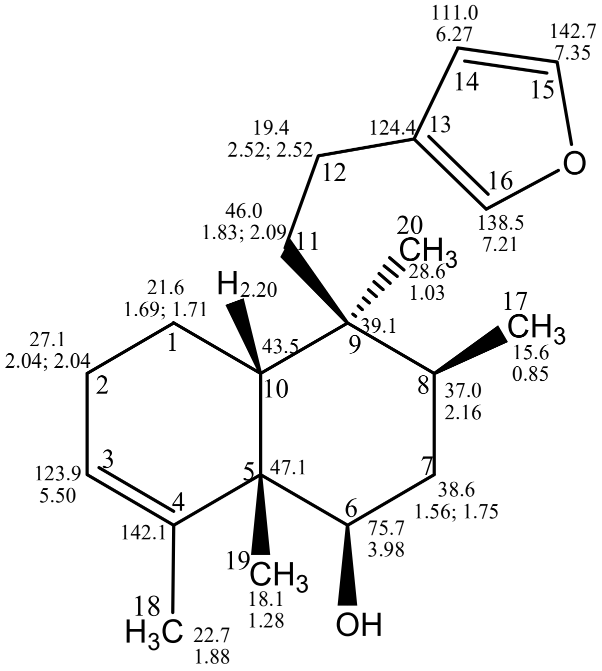

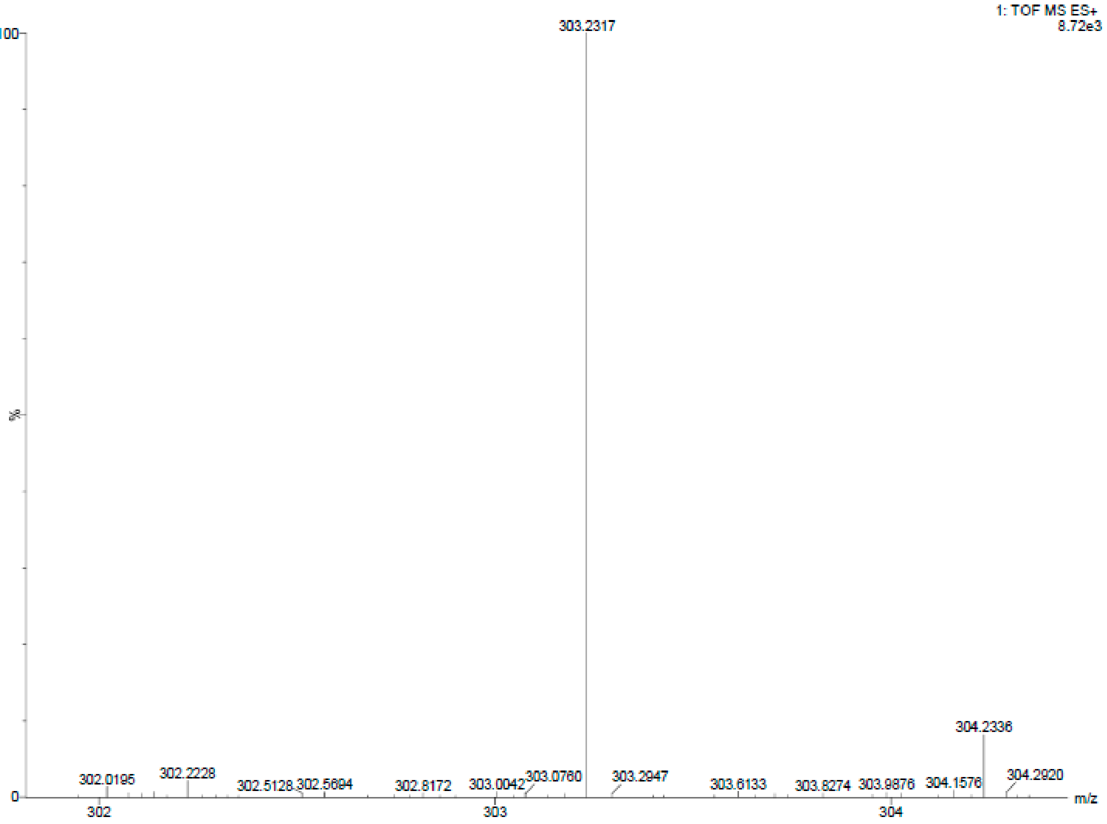

Figure S29: (+)-high-resolution mass spectrum of compound **6**

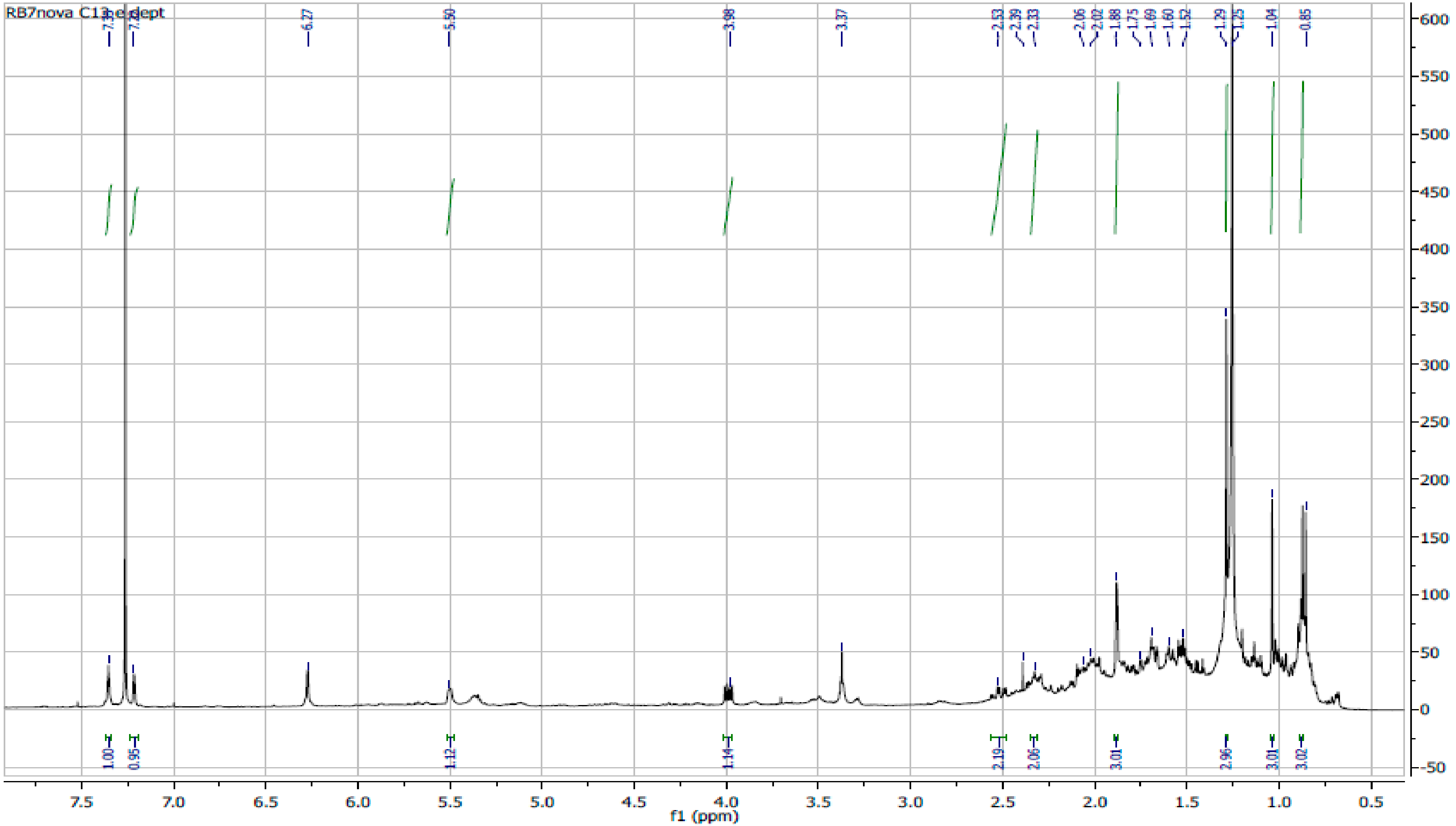

Figure S30: ^{1}H NMR spectrum of compound **6** at 400MHz in $CDCl_3$.

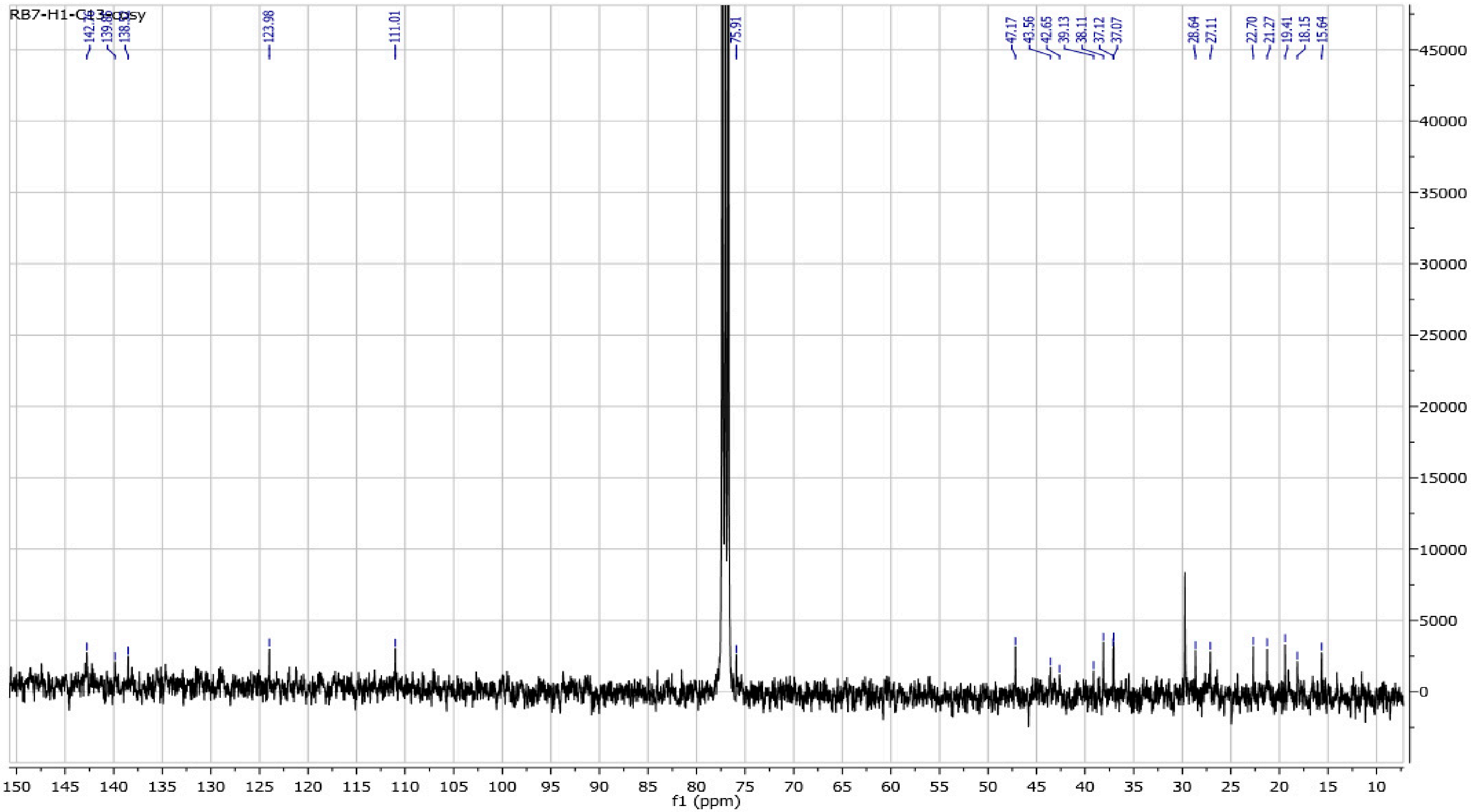

Figure S31: ^{13}C NMR spectrum of compound **6** at 100MHz in $CDCl_3$.

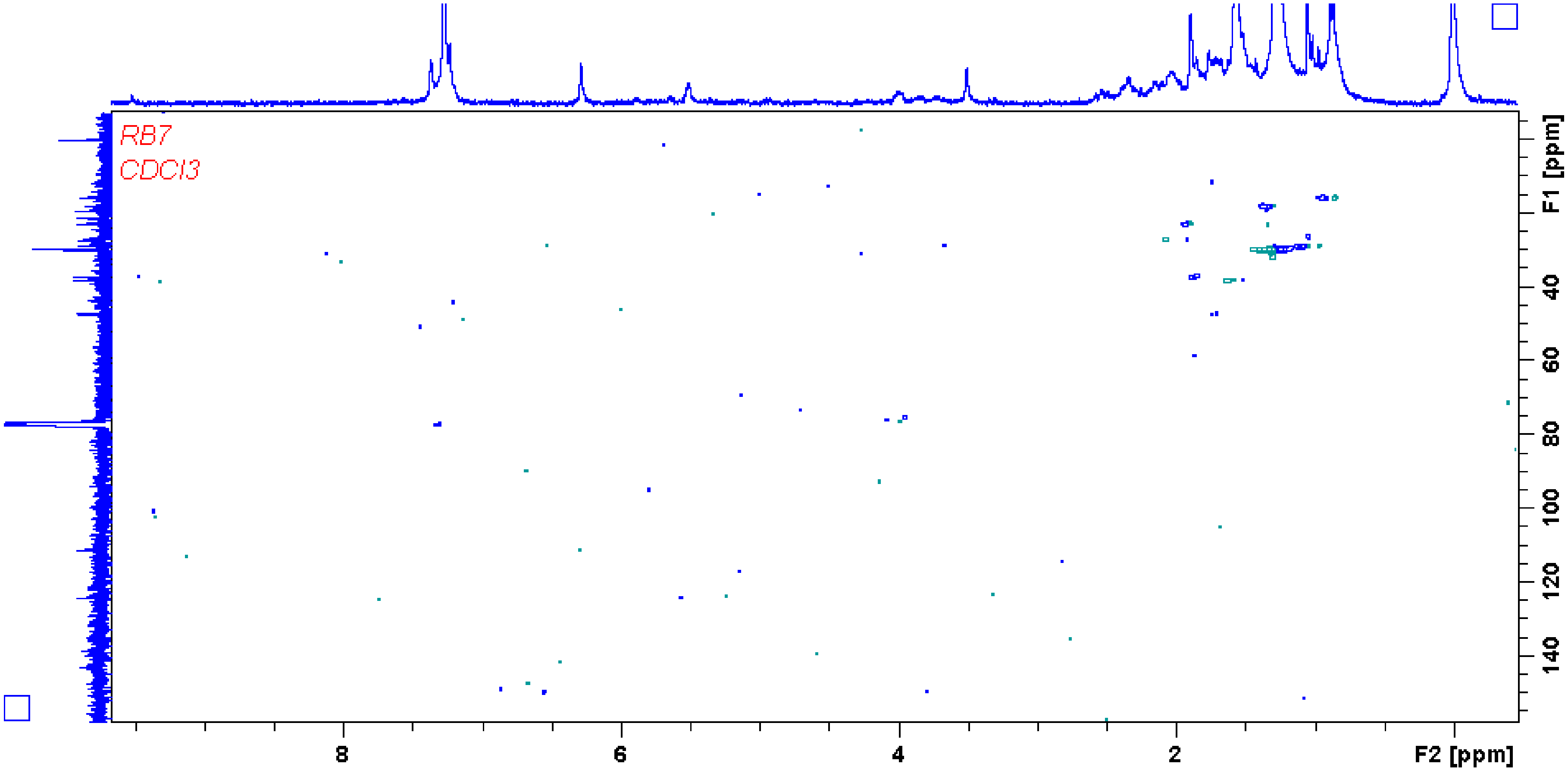

Figure S32: .HSQC NMR spectrum of compound **6** at 300MHz in $CDCl_3$.

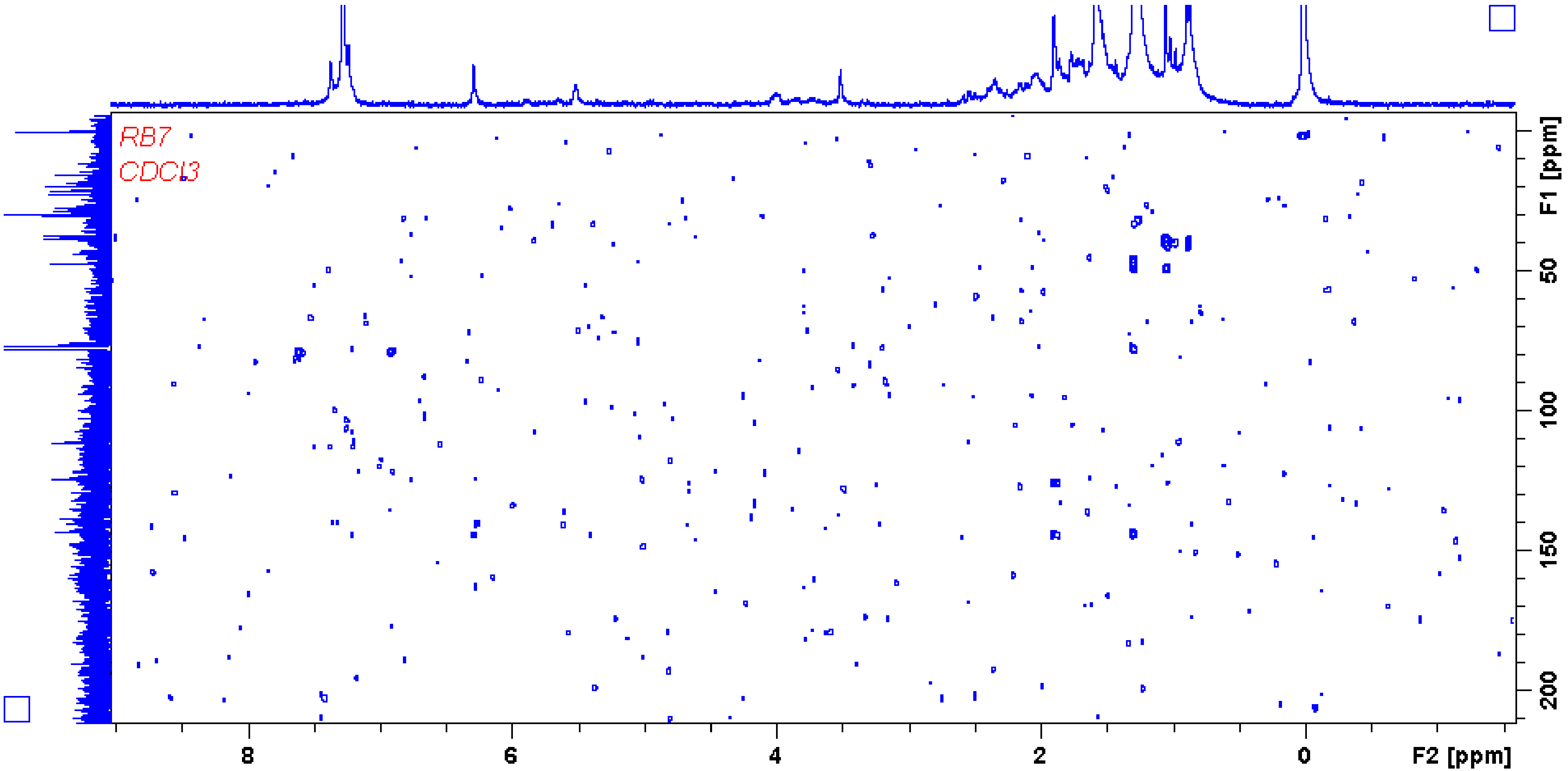

Figure S33: HMBC NMR spectrum of compound **6** at 300 MHz in $CDCl_3$.
